# Pulsed EPR Dipolar Spectroscopy on Spin Pairs with one Highly Anisotropic Spin Center: The Low‐Spin Fe^III^ Case

**DOI:** 10.1002/chem.201902908

**Published:** 2019-10-09

**Authors:** Dinar Abdullin, Philipp Brehm, Nico Fleck, Sebastian Spicher, Stefan Grimme, Olav Schiemann

**Affiliations:** ^1^ Institute of Physical and Theoretical Chemistry University of Bonn 53115 Bonn Germany; ^2^ Current address: Institute of Inorganic Chemistry University of Bonn 53115 Bonn Germany; ^3^ Mulliken Center for Theoretical Chemistry University of Bonn 53115 Bonn Germany

**Keywords:** EPR spectroscopy, iron(III), proteins, spin labels, trityl radicals

## Abstract

Pulsed electron paramagnetic resonance (EPR) dipolar spectroscopy (PDS) offers several methods for measuring dipolar coupling constants and thus the distance between electron spin centers. Up to now, PDS measurements have been mostly applied to spin centers whose *g*‐anisotropies are moderate and therefore have a negligible effect on the dipolar coupling constants. In contrast, spin centers with large *g*‐anisotropy yield dipolar coupling constants that depend on the *g*‐values. In this case, the usual methods of extracting distances from the raw PDS data cannot be applied. Here, the effect of the *g*‐anisotropy on PDS data is studied in detail on the example of the low‐spin Fe^3+^ ion. First, this effect is described theoretically, using the work of Bedilo and Maryasov (*Appl. Magn. Reson*. **2006**, 30, 683–702) as a basis. Then, two known Fe^3+^/nitroxide compounds and one new Fe^3+^/trityl compound were synthesized and PDS measurements were carried out on them using a method called relaxation induced dipolar modulation enhancement (RIDME). Based on the theoretical results, a RIDME data analysis procedure was developed, which facilitated the extraction of the inter‐spin distance and the orientation of the inter‐spin vector relative to the Fe^3+^ *g*‐tensor frame from the RIDME data. The accuracy of the determined distances and orientations was confirmed by comparison with MD simulations. This method can thus be applied to the highly relevant class of metalloproteins with, for example, low‐spin Fe^3+^ ions.

## Graphical abstract

**Pulsed EPR dipolar spectroscopy** is established for highly anisotropic spin centers on example of the biologically relevant low‐spin Fe^III^ ion. This methodology was benchmarked on three model compound, yielding accurate estimates of inter‐spin distances, as well as the orientations of the inter‐spin vector relative to the *g*‐tensor axes of the Fe^III^ ion.

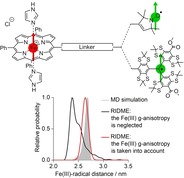

## Introduction

Pulsed EPR dipolar spectroscopy (PDS), which includes techniques such as pulsed electron–electron double resonance (PELDOR or DEER),^1^, ^2^ double quantum coherence EPR (DQC),^3^ single‐frequency technique for refocusing dipolar couplings (SIFTER),^4^ and relaxation induced dipolar modulation enhancement (RIDME),^5^, ^6^ is a valuable method for determining biomolecular structures and their conformational changes during function.^7^ The method is based on the measurement of the dipolar coupling between electron spin centers and provides information about the inter‐spin distances and, in favorable cases, the relative orientation of these centers. As the majority of biomolecules are naturally diamagnetic, PDS on such systems typically requires site directed labeling of the biomolecule with spin labels.^8^, ^9^, ^10^, ^11^, ^12^ The most common spin labels are nitroxides,^9^, ^10^, ^11^, ^12^ although a number of alternatives based on Gd^3+^,^13^, ^14^ Cu^2+^,^15^, ^16^ trityl^17^, ^18^, ^19^, ^20^ and photoexcited porphyrins^21^ have been reported. In addition to the spin labels, there is a keen interest of using naturally occurring paramagnetic cofactors, such as Cu^2+^,^22^, ^23^, ^24^, ^25^, ^26^, ^27^, ^28^ low‐spin (LS) Fe^3+^,^6^, ^29^, ^30^, ^31^, ^32^ high‐spin (HS) Fe^3+^,^33^ HS Mn^2+^,^34^, ^35^, ^36^, ^37^, ^38^ Mo^5+^,^30^ Co^2+^,^39^, ^40^ iron–sulfur clusters,^27^, ^41^, ^42^ manganese clusters,^43^ tyrosins,^44^, ^45^ semiquinones^46^ or flavins,^29^, ^47^, ^48^ for PDS measurements. The obvious advantage of using intrinsic spin centers is that the number of spin labels required for PDS and, consequently, the number of structural perturbations to the native biomolecular structure can be reduced. Moreover, intrinsic spin centers often have a well‐defined, fixed position within the fold of the biomolecule and, thus, can provide more accurate distance constraints as compared to flexible spin labels. In addition, PDS‐based distance measurements between an intrinsic spin center and spin labels at different sites of a biomolecule enable the localization of the intrinsic spin centers within the biomolecular fold through trilateration^49^ or the docking of different parts of protein complexes using paramagnetic metal ions as anchor points.^50^

Low‐spin Fe^3+^ ions occur widely in metalloproteins,^51^, ^52^, ^53^ for example in hemoglobin, myoglobin, or cytochromes, and constitute as such an important spin probe for PDS. As compared to organic radicals and other low‐spin metal centers like Cu^2+^, LS Fe^3+^ ions have a large *g*‐tensor anisotropy and shorter relaxation times, which impose significant challenges on PDS measurements and the corresponding data analysis. Due to the significant *g*‐anisotropy, the spectral width of LS Fe^3+^ ions largely exceeds the bandwidth of typical microwave pulses at usual microwave frequencies. Consequently, when the PDS signal is acquired on the LS Fe^3+^ centers, only a small fraction of these centers contributes to the signal. This, together with the short *T_m_* relaxation rate of the LS Fe^3+^ ions, led to a low signal‐to‐noise ratio (SNR) of the X‐ and Q‐band PELDOR time traces acquired on the LS Fe^3+^/nitroxide spin pair in neuroglobin.^54^ Later, the value of SNR was improved by a factor of 30 using composite pulses at W‐band.^32^ However, even though a reasonable SNR could be achieved, an accurate conversion of the PELDOR time traces into the distance distributions can be obstructed by orientation selectivity effects, due to selective excitation of certain orientations of the LS Fe^3+^ spin. The common way to account for the orientation selectivity is to measure several PELDOR time traces for different orientations of an anisotropic spin and then to analyze all time traces together.^55^, ^56^, ^57^, ^58^, ^59^ However, this procedure is not applicable to the spin pair LS Fe^3+^/nitroxide, because the difference in resonance frequencies between LS Fe^3+^ ions and nitroxides exceeds the bandwidths of EPR resonators and microwave amplifiers.

The difficulties of PELDOR experiments involving LS Fe^3+^/organic radical spin pairs can be circumvented by using another PDS technique called RIDME. The key difference of this technique to PELDOR is that one of the dipolar‐coupled spins is flipped not by a selective microwave pulse, called pump pulse, but by non‐selective spontaneous relaxation events. Since the *T*_1_ relaxation times of organic radicals is typically much longer than the *T*_1_ relaxation times of LS Fe^3+^ ions, the RIDME signal is usually acquired on the organic radicals, whereas the Fe^3+^ spins are flipped by spontaneous relaxation. The experiment done this way has several advantages. First, the detection of the RIDME signal on the organic radical, which has a small *g*‐anisotropy and a long *T_m_* relaxation time, results in a good SNR of the RIDME time trace. Second, an infinite effective bandwidth of the stochastic Fe^3+^ spin flips can provide RIDME modulation depths of up to 50 % and ensures the absence of orientation selectivity from LS Fe^3+^. These advantages were confirmed in previous RIDME studies on LS Fe^3+^/flavin,^29^ LS Fe^3+^/nitroxide^6^, ^31^ and LS Fe^3+^/trityl^20^ spin pairs.

Although acquiring RIDME time traces on LS Fe^3+^/organic radical spin pairs is fairly straightforward, the conversion of these time traces into the distance distributions is challenging. As was pointed out by Milikisyants et al.^6^ and later by Astashkin et al.,^29^ the deviation of the three principal *g*‐values of LS Fe^3+^ ions from the *g*‐factor of the free electron (*g_e_*≈2.0023) is large enough that it cannot be neglected in RIDME data analysis. This means that the common methods of PDS data analysis, which assumes both spins to be almost isotropic, cannot be applied in the present case. Instead, the theory of Bedilo and Maryasov^60^ for the dipolar coupling between anisotropic spins centers has to be used in this case. The first application of this theory for the analysis of RIDME data was reported by Astashkin et al.^29^ There, the RIDME spectrum of the LS Fe^3+^/flavin spin pair was simulated using a modified equation for the dipolar coupling constant, which provided estimates of the inter‐spin distance and two angles that determine the relative orientation of the Fe^3+^ *g*‐tensor with respect to the distance vector. However, this analysis was done only in a semi‐quantitative way, because usage of the four‐pulse RIDME sequence lead to time traces with significant dead time and the SNR was rather low.

**Figure 1:**
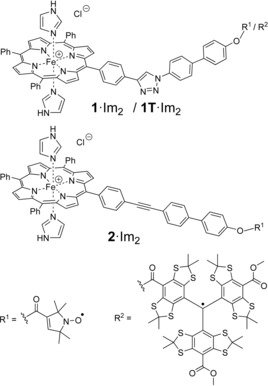
Lewis structures of model compounds **1**⋅Im_2_, **1T**⋅Im_2_ and **2**⋅Im_2_.

The aim of the study here is therefore to explore the effect of *g*‐anisotropy on the RIDME data of LS Fe^3+^/organic radical systems and to establish a quantitative analysis for such data. First, the theory of the dipole–dipole interaction between a LS Fe^3+^ ion and an organic radical is given and then the predictions are derived for the shape of the corresponding dipolar spectra. To confirm the predictions experimentally, two known Fe^3+^/nitroxide compounds, **1** and **2**,^61^ and one new Fe^3+^/trityl compound, **1T**, were synthesized (Figure 1) and RIDME data was acquired on them. The obtained RIDME time traces were analyzed using the program DipFit, which was originally developed for the high‐spin Fe^3+^/nitroxide pairs^33^ but was extended here to the case of LS Fe^3+^. At last, the DipFit‐based distance and angular distributions were compared to the results of molecular dynamics (MD) simulations and to the distance distributions obtained for the same RIDME data by means of the program DeerAnalysis.^62^

## Theory

**Figure 2:**
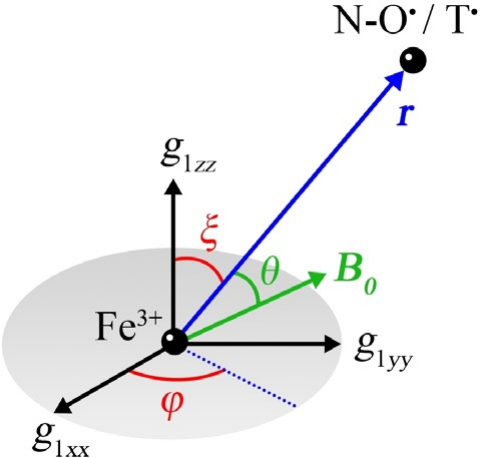
Geometric model of the spin pairs LS Fe^3+^/nitroxide and LS Fe^3+^/trityl in an external magnetic field ***B***_**0**_.

The theory of dipole‐dipole interaction between two anisotropic spin‐1/2 centers was developed by Bedilo and Maryasov^60^ and was later extended to the case of a dipole‐dipole interaction between one anisotropic spin‐1/2 center and one isotropic spin‐1/2 center.^29^, ^33^ The latter case applies for the spin pairs LS Fe^3+^/organic radical, because the *g*‐anisotropy of organic radical has usually little effect on dipolar spectra and thus can be neglected. Therefore, and as shown previously by us,^33^ the dipolar coupling frequency *ν*_dd_ of such spin pairs can be described by Equation (1):

(1) $$\nu_{dd}=\frac{\mu_{0}}{4\pi h}\frac{\beta_{e}^{2}g_{1eff}g_{e}}{r^{3}}\left[1-3\left(\frac{{\hat{g}}_{1}{\hat{g}}_{1}^{T}}{g_{1eff}^{2}}\frac{B_{0}}{B_{0}},\;n\right)\left(\frac{B_{0}}{B_{0}},n\right)\right]$$

in which *μ*_0_ is the vacuum permeability, *h* is the Planck constant, $\hat{g}$ and $g_{eff}$ are the *g*‐tensor and the effective *g*‐factor of the LS Fe^3+^ ion, respectively, ***r*** is the inter‐spin vector with the length *r* and the unit vector ***n***, and ***B***_**0**_ is the vector of applied magnetic field with the length *B*_0_. Note that the product (***B***_**0**_/*B*_0_, ***n***) is usually denoted as *cos*(*θ*), in which *θ* is the angle between the inter‐spin distance vector and the applied magnetic field. Comparing Equation (1) with the equation for the dipolar coupling between two isotropic spin‐1/2 centers [Eq. (2)]:

(2) $$\nu_{dd}^{iso}=\frac{\mu_{0}}{4\pi h}\frac{\beta_{e}^{2}g_{e}^{2}}{r^{3}}\left[1-3{cos}^{2}\left(\theta \right)\right]$$

reveals that both equations differ by the factor *g*_1*eff*_/*g_e_* and the angular term in the square brackets. In the case of two isotropic spins, the latter term depends only on the angle *θ*, whereas when one of the spins is anisotropic, it also depends on the orientation of the distance vector with respect to the *g*‐tensor of the anisotropic spin center. Such orientation can be described by two spherical angles, the polar (*ξ*) and azimuthal (*φ*) angles (Figure 2). Thus, the dipolar coupling frequencies depend not only on *r* and *θ*, but also on the *g*‐values of the anisotropic spin center and the angles *ξ* and *φ*.

**Figure 3:**
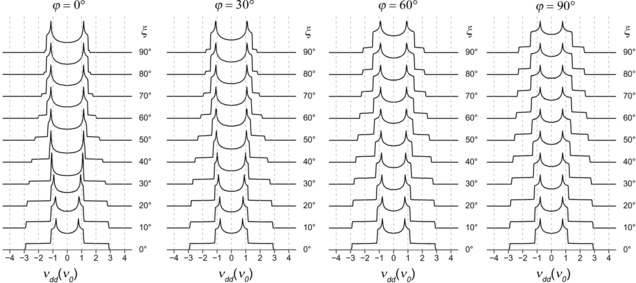
Angular dependence of the dipolar spectrum of a spin pair consisting of an isotropic spin‐1/2 with *g_iso_*=*g_e_* and an anisotropic spin‐1/2 with *g_aniso_*=[1.56, 2.28, 2.91].

To get a deeper insight into how the *g*‐anisotropy of LS Fe^3+^ centers influences the dipolar spectra, spectral simulations were performed on the basis of Equation (1), using *g*_1*xx*_=1.56, *g*_1*yy*_=2.28, *g*_1*zz*_=2.91 for the principal *g*‐values of the low‐spin Fe^3+^ center (they correspond to the experimental *g*‐values discussed below). The *g*‐value of the organic radical was set to *g*_e_=2.0023 and the inter‐spin distance to 2.50 nm. The angles *ξ* and *φ* were varied in the range [0°, 90°] with steps of 10° and 30°, respectively. Averaging of the dipolar coupling frequency over all possible orientations of the spin pair with respect to ***B***_**0**_ was done by the Monte‐Carlo method using 10^6^ random samples. The obtained powder‐averaged spectra are shown in Figure 3. The abscissa of the depicted spectra is given in units derived from Equation (3):

(3) $$\nu_{0}=\frac{\mu_{0}}{4\pi }\frac{\beta_{e}^{2}g_{e}^{2}}{r^{3}}$$

which corresponds to the dipolar coupling constant of an isotropic spin pair with the same inter‐spin distance as used above. Figure 3 reveals a prominent deviation of the calculated spectra from the well‐known Pake doublet. Whereas the Pake doublet has two characteristic singularities, often referred to as perpendicular (*θ*=90°) and parallel (*θ*=0°) components, all calculated spectra here display three singularities instead. In analogy to the Pake doublet, these singularities can be subdivided into two perpendicular components, which correspond to *θ*=90°, and one parallel component, which corresponds to *θ*=0°. Moreover, the frequencies, at which the singularities appear in Figure 3, do not have a fixed ratio, as in the case of the Pake doublet, but depend on the principal *g*‐values of the LS Fe^3+^ center and the angles *ξ* and *φ*. This dependence can be readily explained on example of four spectra corresponding to the angular combinations (*ξ*, *φ*)=(0°, 0°), (0°, 90°), (90°, 0°) and (90°, 90°). For the angular combination (0°, 0°), *r* is collinear to the *g*_1*zz*_‐axis of the LS Fe^3+^ *g*‐tensor. Consequently, the parallel component of the spectrum is scaled by *g*_1*zz*_, yielding a singularity at 2(*g*_1*zz*_/*g_e_*)*ν_0_*≈2.91 *ν_0_*. Then, the other two components of the LS Fe^3+^ *g*‐tensor give rise to two perpendicular components, which appear at (*g*_1*xx*_/*g_e_*)*ν_0_*≈0.78 *ν_0_* and (*g*_1*yy*_/*g_e_*)*ν_0_*≈1.14 *ν_0_*. The same assignment of singularities also holds for the spin pair geometry with (*ξ*, *φ*)=(0°, 90°), because the shape of the dipolar spectrum does not depend on the *φ* angle as soon as the *ξ* angle equals 0°. For the angular combination (90°, 0°), ***r*** is aligned along the *g*_1*xx*_‐axis of the LS Fe^3+^ *g*‐tensor. Thus, the parallel component of the spectrum is scaled by *g*_1*xx*_ and appears at 2(*g*_1*xx*_/*g_e_*)*ν_0_*≈1.56 *ν_0_*, whereas two perpendicular components are scaled by *g*_1*yy*_ and *g*_1*zz*_ and appear at (*g*_1*yy*_/*g_e_*)*ν_0_*≈1.14 *ν_0_* and (*g*_1*zz*_/*g_e_*)*ν_0_*≈1.45 *ν_0_*, respectively. Finally, the angular combination (90°, 90°) corresponds to the case where ***r*** is collinear to the *g*_1*yy*_‐axis of the LS Fe^3+^ *g*‐tensor. In this case, the parallel component of the spectrum is determined by the value of *g*_1*yy*_, which yields the singularity at 2(*g*_1*yy*_/*g_e_*)*ν_0_*≈2.28 *ν_0_*, whereas the perpendicular components of the spectrum are scaled by *g*_1*xx*_ and *g*_1*zz*_ and appear at (*g*_1*xx*_/*g_e_*)*ν_0_*≈0.78 *ν_0_* and (*g*_1*zz*_/*g_e_*)*ν_0_*≈1.45 *ν_0_*.

Note that the dipolar spectra in Figure 3 were simulated for certain values of *r*, *ξ* and *φ*. If the molecule, which hosts the spin pair, has some flexibility, the geometric parameters *r*, *ξ* and *φ* will have some distributions. Obviously, the distributions *P*(*r*), *P*(*ξ*) and *P*(*φ*) will affect the shape and the width the corresponding dipolar spectra. This effect will lead to the averaging the dipolar frequency given by Equation (1) over these distributions. Moreover, a possible correlation between the values of *r*, *ξ* and *φ* might affect the dipolar spectra. Thus, the determination of *P*(*r*), *P*(*ξ*) and *P*(*φ*) from the dipolar spectra is a complex, ill‐posed problem. Nevertheless, a possible algorithm to extract these distributions from the dipolar spectra using several simplifying assumptions is proposed in the Experimental section.

## Experimental Section

### Synthesis of model compounds

**Scheme 1:**
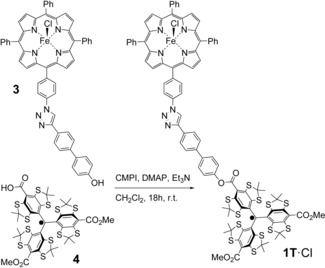
Synthesis of model compound **1T**⋅Cl.

The synthesis and analytics of compounds **1**⋅Cl and **2**⋅Cl have been described previously.^61^ Compound **1T**⋅Cl was obtained according to the following protocol (Scheme 1): First, Fe^3+^‐porphyrin **3** 61 (10 mg, 10.5 μmol), trityl radical **4** 63 (10.8 mg, 10.5 μmol), 2‐chlor‐1‐methylpyridinum iodide (CMPI; 3.2 mg, 12.6 μmol, 1.2 equiv.), and 4‐(*N*,*N*‐dimethylamino)‐pyridin (DMAP; 1.0 mg, 4.2 μmol, 0.4 equiv.) were dissolved in 2 mL dry dichloromethane under argon atmosphere. Then, triethyl amine (2.5 μL, 25.2 μmol, 2.5 equiv.) was added and the reaction mixture was stirred for 18 h at room temperature. Afterwards, water (10 mL) and dichloromethane (10 mL) were added and the organic phase was separated. After washing with brine, the solvent of the organic fraction was removed under reduced pressure and the crude product was subjected to column chromatography. Eluting with dichloromethane/methanol (20:1, v/v), the product was isolated as a dark brownish solid in a yield of 12.9 mg (6.6 μmol; 63 %). For analytical data see Figure S1.

All three compounds were converted from the HS‐ (*S=*5/2) to the LS‐state (*S=*1/2) by adding 10^4^ e^quiv^. of imidazole (Im) to 200 μm solutions of the HS compounds in [D_8_]THF, yielding **1**⋅Im_2_, **2**⋅Im_2_ and **1T**⋅Im_2_, respectively. The spin transition was confirmed by means of cw‐EPR (see below).

### EPR measurements

Details of the experimental setups are described in Chapter 2 of the Supporting Information. The RIDME experiments on the model compounds were performed using the five‐pulse sequence *π*/2−*τ*_1_−*π*−(*τ*_1_+*t*)‐*π*/2−*T_mix_*−*π*/2−(*τ*_2_−*t*)−*π*−*τ*_2_‐*echo*.^6^ The frequency of the microwave pulses was set in resonance with either the maximum of the nitroxide spectrum, for **1**⋅Im_2_ and **2**⋅Im_2_, or with the maximum of the trityl spectrum for **1T**⋅Im_2_. The lengths of the *π*/2 and *π* pulses were 12 and 24 ns, respectively. *τ*_1_ and *τ*_2_ intervals were set to 300 ns and 3 μs, respectively. The optimal values of *T_mix_* and the temperature of RIDME experiments *T* were determined based on the temperature‐dependent inversion recovery measurements on the LS Fe^3+^ center (Chapter 7 in the Supporting Information). This yielded *T_mix_*=100 μs and *T=*10 K for all three model compounds. During the RIDME experiment, *t* was linearly incremented from −50 ns to 2880 ns in increments of 8 ns, yielding 360 data points in total. The shot repetition time was set to 10 ms. To avoid overlap with unwanted echoes with the detected refocused virtual echo, 16‐step phase cycling was employed (Chapter 7 in the Supporting Information). In order to suppress deuterium ESEEM (Chapter 7 in the Supporting Information), the initial values of *τ*_1_ and *τ*_2_ were incremented stepwise and independently from each other with an increment of 8 ns and 16 steps per inter‐pulse interval,^64^ resulting in additional 256 averaging cycles. As a result, the duration of a single RIDME experiment was about 4 hours.

### RIDME data analysis

The first step of the RIDME data analysis was the usual removal of the non‐oscillating background from the original RIDME time traces. According to the recent publication by Keller et al.,^65^ the shape of the RIDME background is described by an exponential function with an argument having a linear and a quadratic terms with respect to *t*. In practice, it is often approximated by a stretched exponential function or a third‐order polynomial function.^31^, ^65^, ^66^ Here, the background was fitted here by a third order polynomial function using the program DeerAnalysis.^62^ The same program was used to divide the original RIDME time traces by the background function and then to perform fast Fourier transformation (FFT) of the background corrected time traces. This procedure yielded the so‐called RIDME spectra.

In the next step, the RIDME time traces were fitted by means of the program DipFit^33^ (free available at https://github.com/dinarabdullin). In this program, the distributions *P*(*r*), *P*(*ξ*) and *P*(*φ*) were approximated by Gaussians and the corresponding mean values ⟨*r*⟩, ⟨*ξ*⟩, ⟨*φ*⟩ and standard deviations Δ*r*, Δ*ξ*, Δ*φ* were used as fitting parameters. All six fitting parameters were optimized until the simulated time trace or the simulated spectrum provided the best root‐mean‐square deviation (RMSD) to the experimental time trace or the experimental spectrum, respectively. The simulation of the dipolar spectrum for certain values of ⟨*r*⟩, ⟨*ξ*⟩, ⟨*φ*⟩, Δ*r*, Δ*ξ*, and Δ*φ* was done by averaging Equation (1) over the corresponding distributions *P*(*r*), *P*(*ξ*) and *P*(*φ*) and, additionally, over the angle θ (powder averaging). The averaging was performed via the Monte‐Carlo method with 10^6^ random samples. The values of *r*, *ξ* and *φ* were assumed to have no correlation with each other. The *g*‐tensor of the LS Fe^3+^ center was set to the experimental values described below. For simplicity, the nitroxide's and trityl's principal *g*‐values were set to *g_e_*. In order to simulate the RIDME time trace, the 10^6^ values of the dipolar frequency were used to compute the sum in Equation (4):^67^

(4) $$\frac{V\left(t\right)}{V\left(0\right)}=1-\frac{\lambda }{N}\sum_{i=1}^{N}\left(1-cos\left(2\pi \nu_{dd}^{i}t\right)\right)\;$$

in which, *N* is the number of Monte‐Carlo samples, and *λ* is the modulation depth parameter, which was determined from the experimental RIDME time traces.

### Molecular modeling

The structure optimization and MD simulations for model compounds **1**⋅Im_2_, **2**⋅Im_2_ and **1T**⋅Im_2_ were done using the stand‐alone program xtb.^68^ Evaluation of the MD trajectories was performed with the program TRAVIS.^69^ Owing to the bi‐radical electronic structure and the large molecular size of the model compounds, the semi‐empirical tight‐binding method GFN2‐xTB/GBSA^70^ was applied (for details see Chapter 11 in Supporting Information).

To enable the comparison of the MD results with the structural information from RIDME, the distributions *P*(*r*), *P*(*ξ*) and *P*(*φ*) were calculated based on the MD trajectories. The values of *r* were determined as the distance between the Fe atom and the center of the N−O bond of the nitroxide radical or the central C atom of the trityl radical. To determine the angular parameters *ξ* and *φ*, the orientation of the LS Fe^3+^ *g*‐tensor relative to tetraphenylporphyrin (TPP) had to be defined. Here, this orientation was set to the one reported for Fe(TPP)(4‐MeIm)_2_^+^.^71^ Thus, the *g_zz_*‐axis of LS Fe^3+^ was orthogonal to the TPP plane and aligned with the Fe‐N(imidazole) bond. The corresponding *g_xx_*‐ and *g_yy_*‐axes were aligned with two orthogonal Fe‐N(porphyrin) bonds within the TPP plane. Based on this definition of the *g*‐axes, *ξ* was determined as the angle between the *g_zz_*‐axis of LS Fe^3+^ and the inter‐spin vector ***r***. The angle between the *g_xx_*‐axis and the projection of ***r*** on the TPP plane yielded the value of *φ*. The resulting MD distributions of *r*, *ξ* and *φ* are summarized in Figure S10.

## Results and Discussion

### Preparation of the model compounds

**Figure 4:**
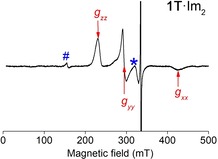
X‐band cw‐EPR spectrum of **1T**⋅Im_2_^+^. The spectrum have been recorded with a microwave frequency of 9.400 GHz at a temperature of 15 K. The spectral positions, which correspond to the principal *g*‐values of the LS Fe^3+^ ion, are marked by arrows. The unsaturated signal of the trityl radicals is given in Figure S3. The cavity background signal is marked by a star, and the hash symbol shows the position of the signal assigned to free Fe^3+^ ions.

The HS‐precursors **1**⋅Cl, **2**⋅Cl and **1T**⋅Cl were synthesized as described in the Experimental part. Adding 10^4^ equivalents of imidazole to the three compounds lead to the formation of the bis‐imidazole adducts **1**⋅Im_2_, **2**⋅Im_2_ and **1T**⋅Im_2_ and conversion of the Fe^3+^ from the HS‐ to the LS‐state. In order to confirm the LS‐state of the Fe^3+^ ions in **1**⋅Im_2_, **2**⋅Im_2_ and **1T**⋅Im_2_, X‐band cw‐EPR spectra of these compounds were measured at 15 K (Figures 4 and S2). The obtained spectra do show the characteristic signal of the LS Fe^3+^ ion, which is overlaid with the sharp saturated signal of the nitroxide or trityl radicals. No HS Fe^3+^ signal, as found for **1**⋅Cl and **2**⋅Cl,^61^ was observed in the region of *g*≈6. Thus, cw‐EPR proofs complete conversion of the HS Fe^3+^ ion into its LS‐state, which is in agreement with previous studies.^72^, ^73^ The principal *g*‐values of the LS Fe^3+^ ion were found to be identical for all three compounds, *g_zz_*=2.91±0.01, *g_yy_*=2.28±0.01 and *g_xx_*=1.56±0.04. All three *g*‐values are in agreement with those reported for the LS Fe^3+^ ion in Fe(TPP)(Im)_2_^+[73]^ and Fe(TPP)(4‐MeIm)_2_^+^.^71^ The spectrum of **1T**⋅Im_2_^+^ contains an additional weak signal at *g*≈4.3, which is assigned to free Fe^3+^ ions that drop out of the porphyrin ring.^74^

### RIDME measurements

**Figure 5:**
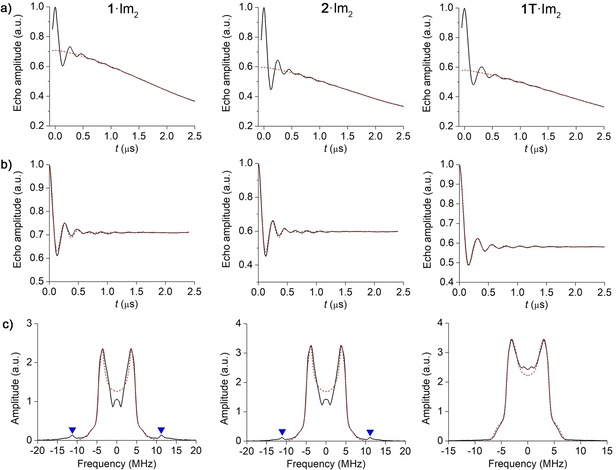
Q‐band RIDME data acquired on **1**⋅Im_2_, **2**⋅Im_2_ and **1T**⋅Im_2_. a) Original RIDME time traces (black solid lines) overlaid with the corresponding third‐order polynomial background fits (red dashed lines). b)  Background‐corrected RIDME time traces and their fits (red dashed lines) obtained by means of DipFit. **c)** FFTs of the RIDME time traces are depicted by the black solid lines. The unsuppressed nitrogen ESEEM peak is marked by the triangles. The dipolar spectra simulated for the optimized distributions *P*(*r*), *P*(*ξ*) and *P*(*φ*) (see Table 1) are depicted by the red dashed lines.

The RIDME time traces recorded on **1**⋅Im_2_, **2**⋅Im_2_ and **1T**⋅Im_2_ are shown in Figure 5 a and the corresponding background‐corrected time traces are depicted in Figure 5 b. All RIDME time traces have a very good SNR (see Chapter 8 in the Supporting Information) and display several clear oscillation periods. The obtained modulation depths equal 30 %, 40 % and 42 % for **1**⋅Im_2_, **2**⋅Im_2_ and **1T**⋅Im_2_, respectively. The difference between the modulation depths, as well as the deviation of these depths from the expected value of 50 %, is likely due to partial μ_2_‐oxo‐dimerization of the Fe^3+^ porphyrins, which was already observed earlier for the given model compounds.^61^

The dipolar spectra corresponding to each of the RIDME time traces are depicted in Figure 5 c. In addition to the dipolar spectra, which appear within ±10 MHz for all three model compounds, the RIDME spectra of **1**⋅Im_2_ and **2**⋅Im_2_ display a weak peak at about 12 MHz. This peak can be assigned to nitroxide ESEEM (Figure S4) and results from incomplete ^14^N ESEEM suppression by the modulation averaging Scheme. Since the amplitude of the unsuppressed ESEEM peak is weak, no further attempts were taken to remove it. Note also that this detrimental ESEEM peak is absent in the RIDME spectrum of the trityl‐based model compound **1T**⋅Im_2_.

### RIDME data analysis

**Figure 6:**
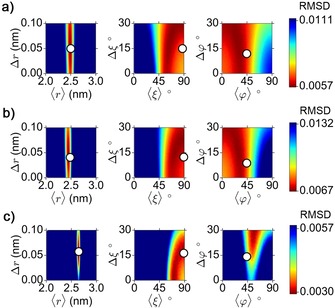
Dependencies of the RMSD between the experimental and simulated RIDME time traces on the mean values and standard deviations of *r*, *ξ* and *φ*. The MD‐based estimates for the mean values and standard deviations of *r*, *ξ* and *φ* are depicted as circles. a) **1**⋅Im_2_, b) **2**⋅Im_2_, and c) **1T**⋅Im_2_.

After the successful acquisition of RIDME data on the model compounds, the next step is the extraction of the distributions *P*(*r*), *P*(*ξ*) and *P*(*φ*) from this data. This was done by means of the program DipFit, which approximates all three distributions by Gaussians and performs the fitting of the RIDME time traces using the mean values and standard deviations of *r*, *ξ* and *φ* as fitting parameters. Figure 5 b shows that good fits to the RIDME time traces were obtained for all three model compounds. The parameters of the distributions *P*(*r*), *P*(*ξ*) and *P*(*φ*), which led to these fits, are listed in Table 1. In order to estimate how defined these parameters are, the six‐dimensional parameter space needs to be explored, which is a very time expensive procedure. Instead, the lower bound for parameters’ uncertainty was determined here by recording the dependence of the goodness of fit on different pairs of fitting parameters, while setting the four other parameters to their optimized values. As a measure for the goodness of fit, the RMSD between the experimental and simulated time traces was used. The obtained RMSD plots for the mean values and the standard deviations of *r*, *ξ* and *φ* are shown in Figure 6, and the RMSD plots for other pairs of fitting parameters are given in Figure S6. Parameter ranges, in which 110 % of the minimal RMSD are reached, were used to determine the approximate confidence intervals for the optimized parameters (Table 1). Note that such error‐estimation criterion is also used in the program PeldorFit, where it was shown to yield reasonable error estimates of fitting parameters.^75^

**Table 1:** RIDME‐ and MD‐based parameters of distributions *P*(*r*), *P*(*ξ*) and *P*(*φ*) in **1**⋅Im_2_, **2**⋅Im_2_ and **1T**⋅Im_2_.

Parameter	**1**⋅Im_2_		**2**⋅Im_2_		**1T**⋅Im_2_	
	RIDME	MD	RIDME	MD	RIDME	MD
⟨*r*⟩ (nm)	2.48±0.03	2.50	2.44±0.02	2.47	2.64±0.01	2.65
Δ*r* (nm)	0.05±0.05	0.05	0.05±0.04	0.04	0.06±0.02	0.06
⟨*ξ*⟩ (°)^[a]^	69±21	86	84±23	90	71±19	88
Δ*ξ* (°)	24±24	15	23±23	12	3±20	16
⟨*φ*⟩ (°)^[a]^	27±27	45	30±30	45	57±7	45
Δ*φ* (°)	20±20	12	13±17	8	30±9	14

[a] These angles were optimized within the range [0°, 90°] but have several symmetry‐related values within the range [0°, 360°] (all sets of symmetry‐related angles are listed in Chapter 9 of the Supporting Information).

As follows from Table 1, the confidence intervals of the mean inter‐spin distance ⟨*r*⟩ and the corresponding standard deviation Δ*r* are well below 1 Å for all three model compounds. Such a precision of the obtained inter‐spin distances reveals high sensitivity of the RIDME experiment towards these two parameters. The confidence intervals of the angular parameters ⟨*ξ*⟩ and Δ*ξ* are also similar for all three compounds. They are in the order of ±20° for both ⟨*ξ*⟩ and Δ*ξ*. The confidence intervals for the angular parameters ⟨*φ*⟩ and Δ*φ* are in average similar to the ones of ⟨*ξ*⟩ and Δ*ξ* but display a larger distribution of their values between different model compounds. The precision of the mean value ⟨*φ*⟩ varies between ±7° (**1T**⋅Im_2_) and ±30° (**2**⋅Im_2_), and the precision of the width Δ*φ* takes values between ±9° (**1T**⋅Im_2_) and ±20° (**1**⋅Im_2_). Such difference in the confidence intervals of ⟨*φ*⟩ and Δ*φ* correlates with the difference in the best RMSD values obtained for each of the model compounds. Figure 6 reveals that the lowest RMSD value among the three model compounds was obtained for **1T**⋅Im_2_, whereas the larger RMSD values were obtained for **1**⋅Im_2_ and **2**⋅Im_2_.

In addition, the optimized distributions *P*(*r*), *P*(*ξ*) and *P*(*φ*) were used to simulate the dipolar spectra in Figure 5 c. As can be seen, the simulated spectra provide an overall agreement with the experimentally obtained spectra. A slight deviation between the experimental and simulated spectra around the zero frequency for **1**⋅Im_2_ and **2**⋅Im_2_ can be due to the imperfection introduced by the background correction. This deviation could not be avoided neither by using various different background fitting functions, such as stretched exponential or polynomial, nor by varying the starting point for the background fitting. Recently, Ritsch et al. have reported on a similar effect observed for a Cu^2+^/nitroxide spin system and assigned this distortion of the RIDME spectrum to the background artifact that appears at the beginning of RIDME time traces.^75^ The ongoing work on the description of the RIDME background might help to explain this empirical observation in the future. Another reason for the observed deviation might be a slight orientation selectivity, which is due to the partial excitation of the nitroxide spectrum in the RIDME experiment.

### Comparison to MD simulations

In order to relate the obtained distributions *P*(*r*), *P*(*ξ*) and *P*(*φ*) to the structure and dynamics of the model compounds, MD simulations were carried out for each of them. Based on these simulations, qualitative estimates of all three distributions were derived (Figure S10) and, to allow direct comparison to the RIDME results, the mean values and their standard deviation were calculated for each distribution. The calculated parameters are listed in Table 1 and are depicted in Figure 6 by circles.

Table 1 reveals an excellent agreement between the RIDME and MD distance parameters ⟨*r*⟩ and Δ*r* for all three model compounds. Both methods predict that **1**⋅Im_2_ and **2**⋅Im_2_ have similar mean Fe^3+^‐nitroxide distances of ∼2.50 nm and ∼2.47 nm, respectively, whereas the mean Fe^3+^‐trityl distance in **1T**⋅Im_2_ is ∼0.15 nm longer than the Fe^3+^‐nitroxide distance in the structurally similar compound **1**⋅Im_2_. The latter difference is due to the larger size of the trityl radical as compared to the nitroxide radical. The widths of the inter‐spin distance distributions Δ*r* are below 0.1 nm and differ between the model compounds by less than 0.02 nm, which reflects a similar flexibility of the linker/nitroxide motifs of **1**⋅Im_2_ and **2**⋅Im_2_ and the linker/trityl motif of **1T**⋅Im_2_.

**Figure 7:**
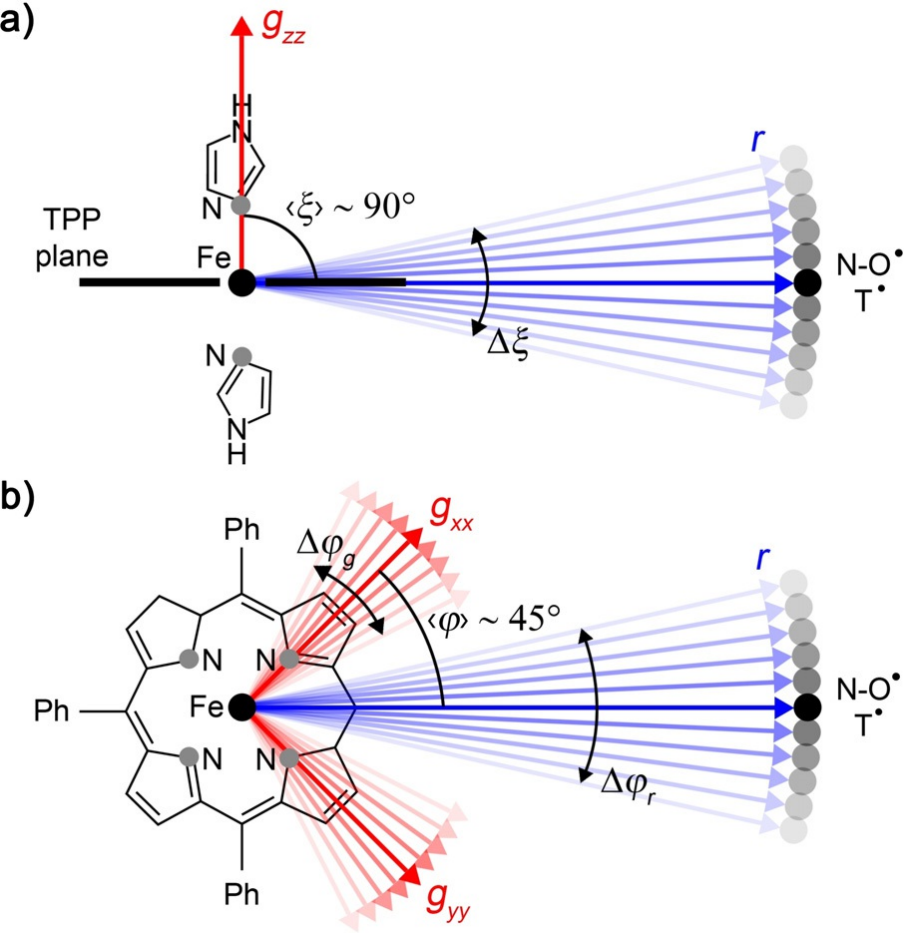
Schematic representation of the geometric parameters ⟨*ξ*⟩ and ⟨*φ*⟩ and their distributions widths Δ*ξ* and Δ*φ* for the model compounds **1**⋅Im_2_, **2**⋅Im_2_ and **1T**⋅Im_2_. The Fe^3+^ and nitroxide (or trityl) spin centers are depicted as black spheres. The inter‐spin vector is shown by blue vector. The *g*‐axes of the Fe^3+^ ions are depicted as red vectors. a) The view is set parallel to the TPP plane. The TPP core is drawn as a black bar. b) The view is set perpendicular to the TPP plane. Δ*φ_r_* denotes the contribution to Δ*φ* which is due to the dynamics of the linker/radical motifs, and Δ*φ_g_* denotes the contribution to Δ*φ* which stems from the distribution of *g_xx_*‐ and *g_yy_*‐orientations within the TPP plane.

A good agreement between RIDME and MD was achieved not only for the distance parameters but also for the angular parameters ⟨*ξ*⟩ and Δ*ξ*. For all three model compounds, MD simulations yielded ⟨*ξ*⟩ and Δ*ξ* values of ∼90° and ∼15°, respectively. Both values are within the confidence intervals of the corresponding parameters determined by RIDME (see Table 1 and Figure 6). Note that ⟨*ξ*⟩=90° describes the case where the inter‐spin vector is perpendicular to the *g*_zz_‐axis of the Fe^3+^ ion. As *g*_zz_ is orthogonal to the TPP plane,^71^ the inter‐spin vector has to be in plane with the TPP ring (Figure 7 a). In terms of structure, this means that the linker/nitroxide and linker/trityl motifs are in plane with the TPP ring. The distribution of the *ξ* angles around 90° with a standard deviation Δ*ξ* ∼15° can be attributed to bending dynamics of the linkers, which leads to a slight inclination of the linker/nitroxide and linker/trityl motifs relative to the TPP plane (Figure 7 a). This dynamics fits to the observed dynamics of other compounds with similar linker groups.^25^, ^26^, ^57^

The MD derived value of ⟨*φ*⟩ is for all three model compounds 45°. This value is within the confidence intervals of the RIDME derived values of ⟨*φ*⟩ for **1**⋅Im_2_ and **2**⋅Im_2_ and deviates by 5° from the RIDME derived value of ⟨*φ*⟩ for **1T**⋅Im_2_. Similarly, the difference between the RIDME and MD estimates of Δ*φ* depends on the model compound. In the case of **1**⋅Im_2_ and **2**⋅Im_2_, the RIDME values of Δ*φ* have large confidence intervals (see Figure 6), which include the MD prediction. In the case of **1T**⋅Im_2_, the RIDME value of Δ*φ* is well‐defined and deviates by at least 7° from the corresponding MD prediction. A possible explanation for this difference can be based on the following: The angle *φ* is determined by the orientations of the *g_xx_*‐ and *g_yy_*‐axes of the Fe^3+^ ion and the orientation of the inter‐spin vector. In the MD simulations, the orientations of the *g_xx_*‐ and *g_yy_*‐axes were fixed along two orthogonal Fe−N bonds of the TPP ring. Thus, the MD values of Δ*φ* are determined only by the dynamics of the linker/nitroxide or linker/trityl motifs, which changes in the orientation of the inter‐spin vector relative to the fixed *g*‐axes. In addition, the distribution of *g_xx_*‐ and *g_yy_*‐orientations within the TPP plane might also contribute to Δ*φ*. According to previous works, such distribution can be caused by rotation of the axial ligands relative to the TPP plane.^71^, ^76^ If one takes this distribution into account, the RIDME width Δ*φ* can be represented as a superposition of a width Δ*φ_r_*, which is due to the dynamics of the linker/radical motifs, and a width Δ*φ_g_*, which stems from the distribution of *g_xx_*‐ and *g_yy_*‐orientations within the TPP plane and which is caused by the rotation of the axial ligands (Figure 7 b). If both contributions are approximated by Gaussians with standard deviations Δ*φ_r_* and Δ*φ_g_*, the total width is given by Δ*φ* ^2^ *=*Δ*φ_r_* ^2^+Δ*φ_g_* ^2^. Assuming that Δ*φ* and Δ*φ_r_* can be associated with the RIDME‐ and MD‐based widths, respectively, Δ*φ_g_*=26° can be determined for **1T**⋅Im_2_. Note that a similar distribution of the *g_xx_*‐ and *g_yy_*‐orientations around the porphyrin's Fe−N bonds was reported for Fe(TPP)(4‐MeIm)_2_^+^; ±25° based on proton HYSCORE experiments.^71^

### Comparison with 2⋅Cl

As the molecular skeleton of **2**⋅Cl is the same as for **2**⋅Im_2_, both compounds differ only with respect to their spin states, HS for the former and LS for the latter. It is thus of interest to compare the geometric parameters obtained from their RIDME data. The RIDME measurements on HS **2**⋅Cl were reported in our previous paper.^33^ There, it was not possible to determine the *P*(*φ*) distribution, because the *g*‐tensor of the HS Fe^3+^ ion had axial symmetry. In analogy to the RIDME‐based distributions here, the distributions *P*(*r*) and *P*(*ξ*) of the HS compound were described by the mean values, ⟨*r*⟩=2.52±0.03 nm and ⟨*ξ*⟩=89°±4°, and the standard deviations, Δ*r*=0.06±0.05 nm and Δ*ξ*=6°±3°, respectively. These values reveal a good overall agreement with the corresponding values for **2**⋅Im_2_ (see Table 1). Thus, these results show the consistency between the distances and angles *ξ* determined for the LS Fe^3+^/nitroxide and the HS Fe^3+^/nitroxide.

### Comparison to DeerAnalysis

**Figure 8:**
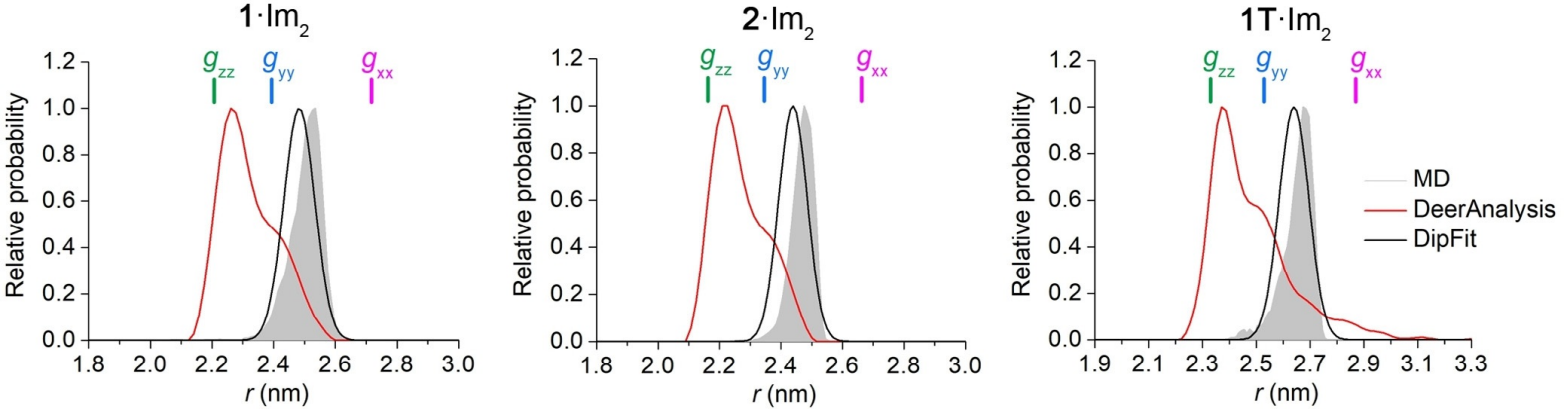
Comparison of DipFit (black lines) and DeerAnalysis (red lines) based inter‐spin distance distributions for **1**⋅Im_2_^+^, **2**⋅Im_2_^+^ and **1T**⋅Im_2_^+^. As a reference, the MD predictions of the distance distributions are depicted as gray shades. Colored bars depict the positions of artificial distances that are obtained for the actual distance ⟨*r*⟩ and three principal *g*‐values of the LS Fe^3+^ center when the *g*‐anisotropy of the Fe^3+^ center is neglected in the RIDME data analysis.

To reveal the effect of the *g*‐anisotropy on the RIDME data analysis, the RIDME time traces of **1**⋅Im_2_, **2**⋅Im_2_ and **1T**⋅Im_2_ were additionally analyzed by means of the program DeerAnalysis,^62^ which neglects the anisotropy of the LS Fe^3+^ spin centers. Figure 8 depicts the distance distributions obtained by DeerAnalysis for all three model systems. For the sake of comparison, these distributions are overlaid with the corresponding DipFit and MD distance distributions. As can be seen from Figure 8, the DeerAnalysis distributions have a clear difference to the DipFit and MD distributions. This difference concerns the most probable distances, which are smaller by ∼0.25 nm for the DeerAnalysis distributions than for the DipFit and MD distributions, as well as the shape of the distance distributions, which are sharp and unimodal in the case of DipFit and MD but are broad and have several prominent shoulders in the case of DeerAnalysis. To interpret the obtained deviation of the DeerAnalysis distance distributions from the corresponding DipFit and MD distributions, it is sufficient to consider Equations (1) and (2). Neglecting their angular parts and putting these two equations equal to each other, than each distance *r* in Equation (1) will correspond to the distance *r*′=*r*⋅(*g_e_/g*_1*eff*_)^1/3^ in Equation (2). This result shows that each actual distance *r* corresponds to a number of artificial distances *r*′ in the DeerAnalysis distribution. Colored bars in Figure 8 depict the positions of these artificial distances calculated for the DipFit parameter ⟨*r*⟩ (Table 1) and the three principal *g*‐values of the LS Fe^3+^ ion (*g_xx_*, *g_yy_*, and *g_zz_* in Figure 4). Since *g_zz_* (=2.91) and *g_yy_* (=2.28) are larger than *g_e_*, the corresponding artificial distances are smaller than the actual distances. In contrast, *g_xx_* (=1.56) is smaller than *g_e_* and the corresponding artificial distance is larger than the actual distance. Thus, the *g*‐anisotropy of Fe^3+^ leads to a shift of the DeerAnalysis distances relative to the actual distances, and the different principal *g*‐values of Fe^3+^ give rise to several shoulders in the DeerAnalysis distributions. In order to predict the exact values of the artificial distances and their relative probabilities, the angular terms of Equations (1) and (2) have to be taken into account as well. This will lead to an additional dependence of the artificial distances on angles *ξ* and *φ*, which are included in the angular term of Equation (1). The dependence of the DeerAnalysis distributions on *ξ* and *φ* is responsible for the fact that the shoulders in the DeerAnalysis distributions appear at not exactly the calculated artificial distances and that these shoulders have different relative probabilities (see Figure 8).

**Figure 9:**
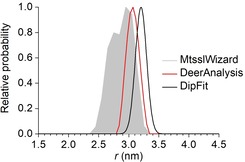
Comparison of DipFit (black lines) and DeerAnalysis (red lines) based inter‐spin distance distributions for the cytochrome P450cam mutant C58R1. As a reference, the mtsslWizard prediction of the distance distribution is depicted as a gray shade.

Thus, the *g*‐anisotropy of the LS Fe^3+^ ion has a significant effect on the RIDME data analysis. Because of this, we revisited the analysis of the RIDME data, reported earlier for the cytochrome P450cam mutant C58R1^31^ (see Chapter 10 in the Supporting Information). Although the *g*‐anisotropy of the LS Fe^3+^ center is smaller for cytochrome P450cam (*g_xx_*=1.91, *g_yy_*=2.25, *g_zz_*=2.42)^31^ then for the model compounds here, a clear difference between the DipFit and DeerAnalysis distance distributions was obtained (Figure 9). This difference concerns mostly the most probable distance, which is smaller by 0.15 nm for the DeerAnalysis distribution as compared to DipFit distribution. The width of the DeerAnalysis distribution is only slightly larger than the width of the DipFit distribution, which is in agreement with the reduced *g*‐anisotropy mentioned above. The DipFit derived distance distribution agrees less well with the MtssWizard^77^, ^78^ prediction than the DeerAnalysis one. However, the difference is close the average error of MtssWizard (∼0.3 nm) and, thus, can be assigned to the uncertainty of in silico prediction.

## Conclusions

The effect of the *g*‐anisotropy of LS Fe^3+^ ions on the RIDME data was described in detail and confirmed experimentally for LS Fe^3+^/nitroxide and LS Fe^3+^/trilyl spin pairs. The dipolar spectra of such spin pairs were shown to depend not only on the inter‐spin distances but also on the principal *g*‐values of LS Fe^3+^ and the relative orientation of the inter‐spin vector relative to the *g*‐frame of Fe^3+^. The latter orientation was described by two angular parameters, a polar angle *ξ* and an azimuthal angle *φ*. The distance distribution *P*(*r*) and the angular distributions *P*(*ξ*) and *P*(*φ*) could be extracted from the experimental RIDME data with the fitting program DipFit. In contrast, the analysis of the same RIDME data using the program DeerAnalysis, which neglects *g*‐anisotropy, led to an error of 0.25 nm in the mean inter‐spin distances, compared to 0.01 nm in the case of DipFit, and errors in the distribution width and shape. In addition, to the distance parameters, DipFit yielded the mean values and standard deviations of angular parameters *ξ* and *φ* with an average uncertainty of 20°. The comparison of the RIDME‐derived distributions *P*(*r*), *P*(*ξ*) and *P*(*φ*) with the their MD‐based predictions revealed very good consistency for all three model systems considered. This result proves that not only *P*(*r*) but also *P*(*ξ*) and *P*(*φ*) can be reliably determined from the RIDME data. Thus, this work provides an important guideline for further applications of PDS to the highly relevant class of LS Fe^3+^ containing proteins and extends the arsenal of available programs for the analysis of PDS data from anisotropic spin centers.

## Conflict of interest

The authors declare no conflict of interest.

## Supporting information

As a service to our authors and readers, this journal provides supporting information supplied by the authors. Such materials are peer reviewed and may be re‐organized for online delivery, but are not copy‐edited or typeset. Technical support issues arising from supporting information (other than missing files) should be addressed to the authors.

Supplementary
