# Supplementary material for: Pulsed EPR Dipolar Spectroscopy on Spin Pairs with one Highly Anisotropic Spin Center: The Low‐Spin FeIII Case
Source: Chemistry. 2019 Oct 9;25(63):14388–98. doi: 10.1002/chem.201902908 (PMC6900076; doi:10.1002/chem.201902908)
Supplement: Supplementary file 1 — Supplementary [file CHEM-25-14388-s001.pdf]

CHEMISTRY
A European Journal

Supporting Information

Pulsed EPR Dipolar Spectroscopy on Spin Pairs with one Highly Anisotropic Spin Center: The Low-Spin Fe^{III} Case

Dinar Abdullin,[a] Philipp Brehm,[a, b] Nico Fleck,[a] Sebastian Spicher,[c] Stefan Grimme,[c] and Olav Schiemann*[a]

chem_201902908_sm_miscellaneous_information.pdf

Table on contents

1 Analytics for compound **1T**·Cl 3

2 Experimental setup for EPR measurements 4

2.1 cw-EPR measurements 4

2.2 Pulsed EPR measurements 4

3 cw-EPR spectra of the ls Fe^{3+} center in **1**·Im2, **2**·Im2 and **1T**·Im2 5

4 cw-EPR spectra of the organic radicals in **1**·Im2, **2**·Im2 and **1T**·Im2 6

5 ESEEM experiments on nitroxide and trityl centers of **1**·Im2, **2**·Im2 and **1T**·Im2 8

6 Inversion recovery experiments on the ls Fe^{3+} center of **1**·Im2, **2**·Im2 and **1T**·Im2 10

7 RIDME experiments on **1**·Im2, **2**·Im2 and **1T**·Im2 11

7.1 Choosing the value of T_{mix} 11

7.2 ESEEM suppression scheme 11

7.3 Phase cycling 12

8 Signal-to-noise ratio of RIDME time traces 13

9 DipFit analysis of the RIDME time traces of **1**·Im2, **2**·Im2 and **1T**·Im2 14

10 DipFit analysis of the RIDME time trace of the P450cam mutant C58R1 16

11 MD simulations for **1**·Im2, **2**·Im2 and **1T**·Im2 18

12 Optimized structure of **1**·Im2 21

13 Optimized structure of **2**·Im2 25

14 Optimized structure of **1T**·Im2 29

References 35

1 Analytics for compound 1T·Cl

Electrospray ionization (ESI+) mass spectra were measured using an OrbitrapXL (Thermo Fisher Scientific) instrument. Gel permeation chromatography (GPC) was conducted on an Agilent 1200 Series analytical GPC system running isocratically with unstabilized THF at a flow rate of 1 ml/min. The system was equipped with polystyrene column (8 mm x 300 mm, four-column set, porosity 10 nm, 100 nm, 10000 nm, and 100000 nm) purchased from PSS Polymer Standard Service GmbH, Germany. The sample was dissolved in unstabilized THF containing 0.1 % trifluoroacetic acid in order to cleave μ_2-oxo dimers.

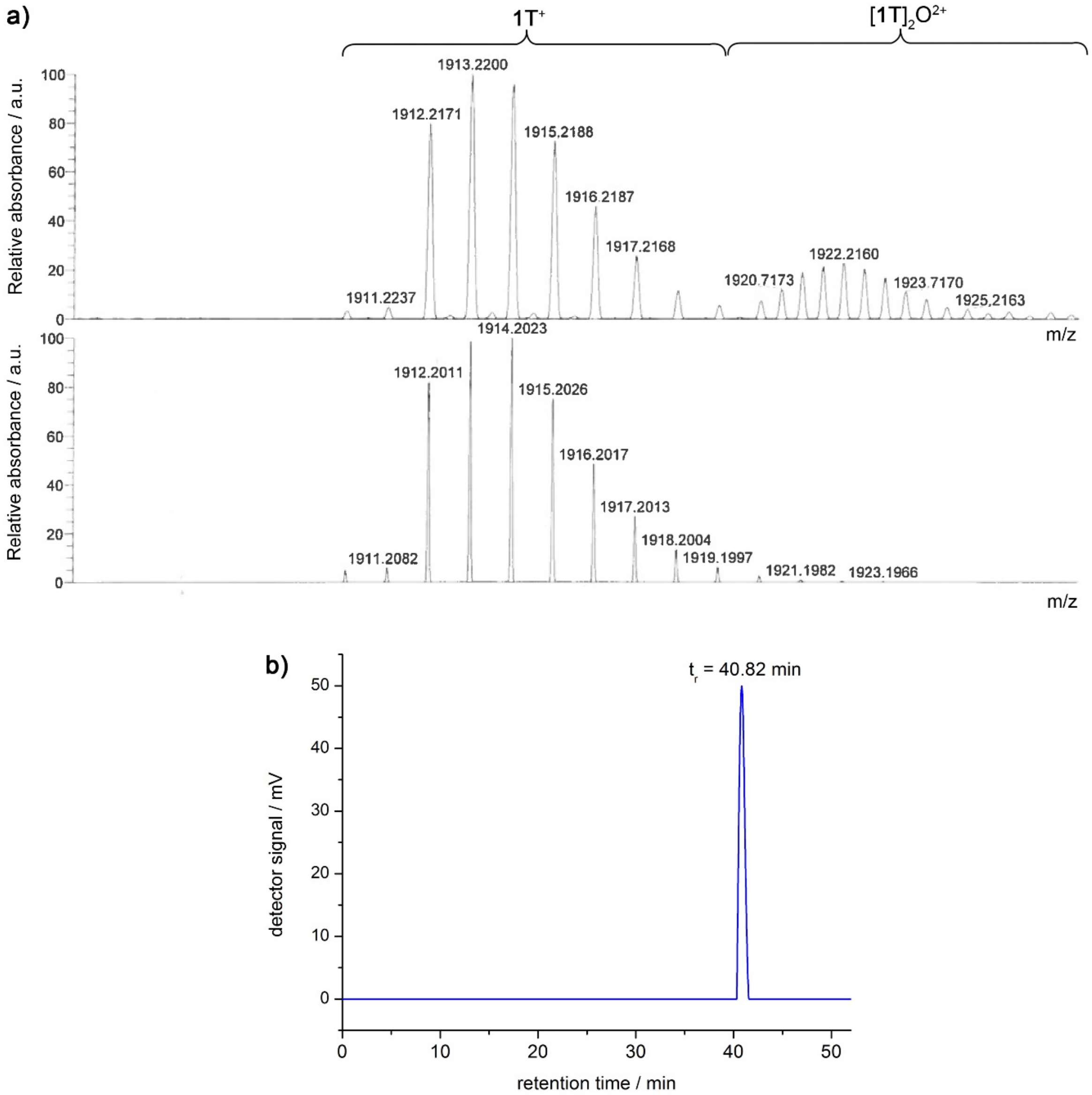

Figure S1. a) The experimental (top) and simulated (bottom) HRMS ESI+ spectrum of **1T**·Cl. Since the spectrum was measured before the HCl-treatment, it reveals the presence of μ_2-oxo-dimers in the sample. b) GPC-elugram of **1T**·Cl.

2 Experimental setup for EPR measurements

2.1 cw-EPR measurements

Continuous wave EPR (cw-EPR) measurements were carried out using a X-band EPR spectrometer EMXmicro (Bruker) equipped with a super high quality resonator (SHQ). The resonator was mounted onto a continuous flow helium cryostat ER4112HE (Bruker), whose temperature was controlled by a Mercury iTC system (Oxford Instruments). To obtain the temperature of 100 K, a nitrogen-based variable temperature accessory ER 4131VT (Bruker) was employed.

2.2 Pulsed EPR measurements

Pulsed EPR measurements were carried out on a Bruker ELEXSYS E580 spectrometer using a Flexline probe head with a Q-band resonator ER5106QT-2 (Bruker). Microwave pulses were amplified with a 150 W TWT amplifier (model 187Ka). In order to obtain temperatures around 10 K, the resonator was mounted inside a continuous flow helium cryostat CF935 (Oxford Instruments) and the temperature inside the cryostat was adjusted by a temperature control system ITC 503 (Oxford Instruments).

3 cw-EPR spectra of the ls Fe^{3+} center in **1**·Im2, **2**·Im2 and **1T**·Im2

The cw-EPR spectra of the ls Fe^{3+} center in **1**·Im2, **2**·Im2 and **1T**·Im2 were acquired at 15 K with a microwave power of 5.529 mW (15 dB), a modulation frequency of 100 kHz, a modulation amplitude of 0.4 mT, and a time constant of 10.24 ms. The central field was set to 255.0 mT, the sweep width to 500.0 mT, the field step to 0.1 mT, and the number of averages to 4.

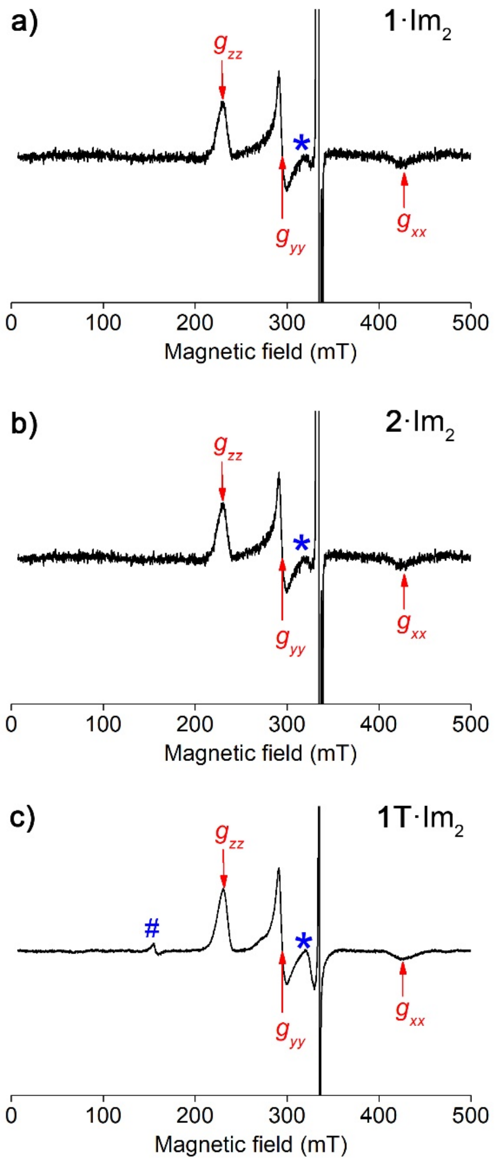

Figure S2. X-band cw-EPR spectra of **a) 1**·Im_2^+, **b) 2**·Im_2^+, and **c) 1T**·Im_2^+. All spectra have been recorded with a microwave frequency of 9.400 GHz at a temperature of 15 K. The spectral positions, which correspond to the principal *g*-values of the ls Fe^{3+} ion, are marked by arrows. The values of g_{zz} and g_{yy} could be readily determined from all three spectra. In contrast, the signal corresponding to g_{xx} is fairly weak and broad. Thus, in order to determine the exact value of g_{xx}, the cw-EPR spectrum of **1T**·Im_2^+ was measured with a sample concentration of 500 µM instead of 200 µM. The unsaturated signals of the organic radicals are given in Figure S3. The cavity background signal is marked by a star, and the hash symbol shows the position of the signal assigned to free Fe^{3+} ions.

4 cw-EPR spectra of the organic radicals in **1**·Im_2, **2**·Im_2 and **1T**·Im_2

The cw-EPR spectra of the nitroxide center in **1**·Im_2 and **2**·Im_2 and the trityl center in **1T**·Im_2 were acquired at 100 K with a microwave power of 1.937 mW (20 dB), a modulation frequency of 100 kHz, a modulation amplitude of 0.1 mT (nitroxide) or 0.01 mT (trityl), and a time constant of 10.24 ms. The microwave frequency was around 9.450 GHz. The central field was set to 336.6 mT, the sweep width to 20 mT, the field step to 0.1 mT, and the number of averages to 1.

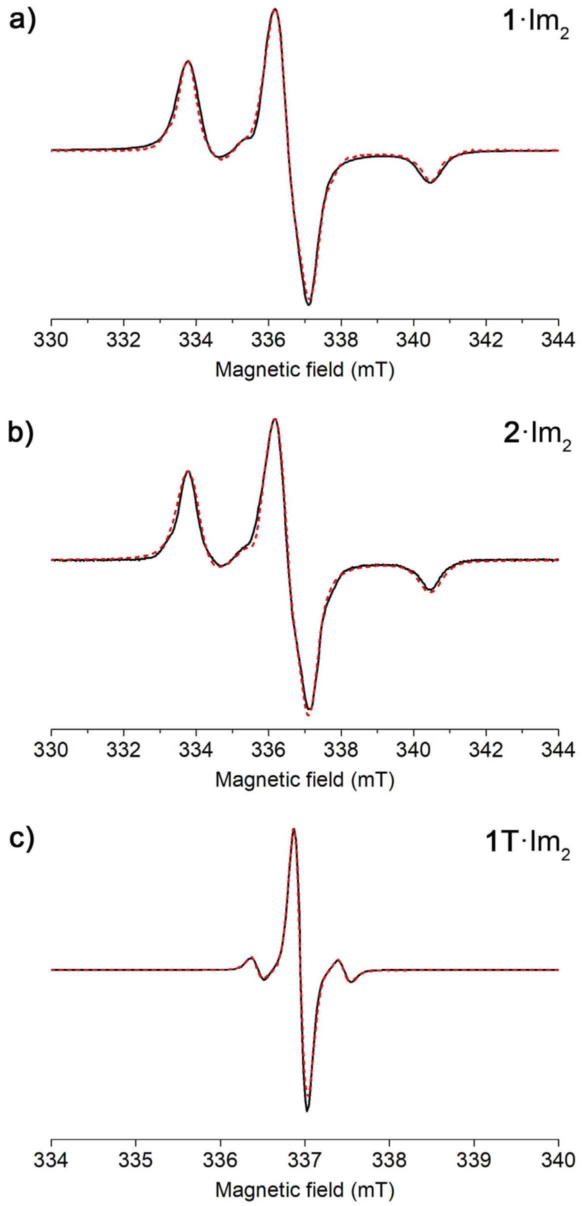

Figure S3. X-band cw-EPR spectra (black solid lines) and their simulations (red dashed lines) for **a)** the nitroxide center of **1**·Im_2, **b)** the nitroxide center of **2**·Im_2 and **c)** the trityl center of **1T**·Im_2. All spectra have been recorded with a microwave frequency of 9.450 GHz at a temperature of 100 K. The simulations were done by means of the program EasySpin.[1] The parameters of the simulations are listed in Table S1.

Table S1. EPR parameters of the nitroxide and trityl spin centers in **1**·Im_2, **2**·Im_2 and **1T**·Im_2.

**Parameter**	**1**·Im_2	**2**·Im_2	**1T**·Im_2
g	[2.0096±0.001, 2.0068±0.001, 2.0027±0.001]	[2.0096±0.001, 2.0068±0.001, 2.0027±0.001]	2.0038±0.001
$A(^{14}N)$ / MHz	[12±6, 12±6, 94±1]	[12±6, 12±6, 94±1]	-
$A(^{13}C)$ / MHz	-	-	29±1
A-strain[a] / MHz	[0, 0, 12]	[0, 0, 12]	-
ΔH_{pp}[b] / mT	[0.26 0.24]	[0.26 0.24]	[0.14 0.04]

[a] For definition see: http://easyspin.org/documentation/broadenings.html

[b] The broadening is described by a Voigt line shape.

5 ESEEM experiments on nitroxide and trityl centers of **1**·Im_2, **2**·Im_2 and **1T**·Im_2

Electron spin echo envelop modulation (ESEEM) measurements were carried out using the pulse sequence *π/2-t-π-t-echo*. The lengths of the *π*/2- and *π*-pulse were 12 and 24 ns, respectively. The initial value of *t* was set to 300 ns and, in course of the experiment, was incremented 2048 times with a constant step of 8 ns. The short repetition time was 1 ms. To suppress unwanted echoes, 2-step phase cycling was performed. For the nitroxide-based compounds **1**·Im_2 and **2**·Im_2, the ESEEM time traces were recorded at three different field positions, called here as positions A, B, and C (Figure S4a). Position A corresponds to the maximum of the nitroxide spectrum and contains contributions from all three components of the ^{14}N hyperfine tensor. Note that this position was also used for the detection of the RIDME time traces. In contrast, position C is selective for the A_{zz} component of the ^{14}N hyperfine tensor. Position B represents an intermediate case between positions A and C. For the trityl-based compound **1T**·Im_2, the ESEEM time traces was recorded only at the maximum of the trityl spectrum, denoted as position D in Figure S4a. For each the samples, the temperature of the ESEEM experiments was set to be the same as the temperature of the corresponding RIDME experiment.

The ESEEM time traces and spectra of all three model compounds are depicted Figure S4b. The corresponding ESEEM spectra are shown in Figure S4c. All spectra in Figure S4c display the distinct peaks at about 7.8 and 15.6 MHz. These peaks can be assigned to the single and double Larmor frequency of deuterium, which is present in a large amount in the deturated solvent. In addition to the deuterium ESEEM, the nitroxide-based model compounds **1**·Im_2 and **2**·Im_2 display one more peak whose frequency depends strongly on the detection position. It appears at 10.8 MHz and 9.6 MHz for the positions A and B, respectively, but it is absent in the spectrum for the position C. Such behavior suggest that this peak should originate from the ^{14}N nucleus of the nitroxide center, whose ESEEM frequency is determined by a combination of the ^{14}N Larmor frequency, the ^{14}N hyperfine coupling constant and the ^{14}N quadrupole coupling constant.[2] Since the value of the ^{14}N hyperfine coupling constant is orientation dependent, it will yield different ESEEM frequencies for different detection positions. For the *x*- and *y*-components of the ^{14}N hyperfine tensor, which are in the order of 7-12 MHz, such combination yields an observable peak at around 10.8 MHz (positions A and B). In contrast, the *z*-component of the ^{14}N hyperfine tensor (~ 93 MHz) is so large that no ESEEM peak can be seen (position C). Note that this nitrogen ESEEM peak was already reported previously for the model compound **2**·Cl and also for the reference nitroxide compound.[3]

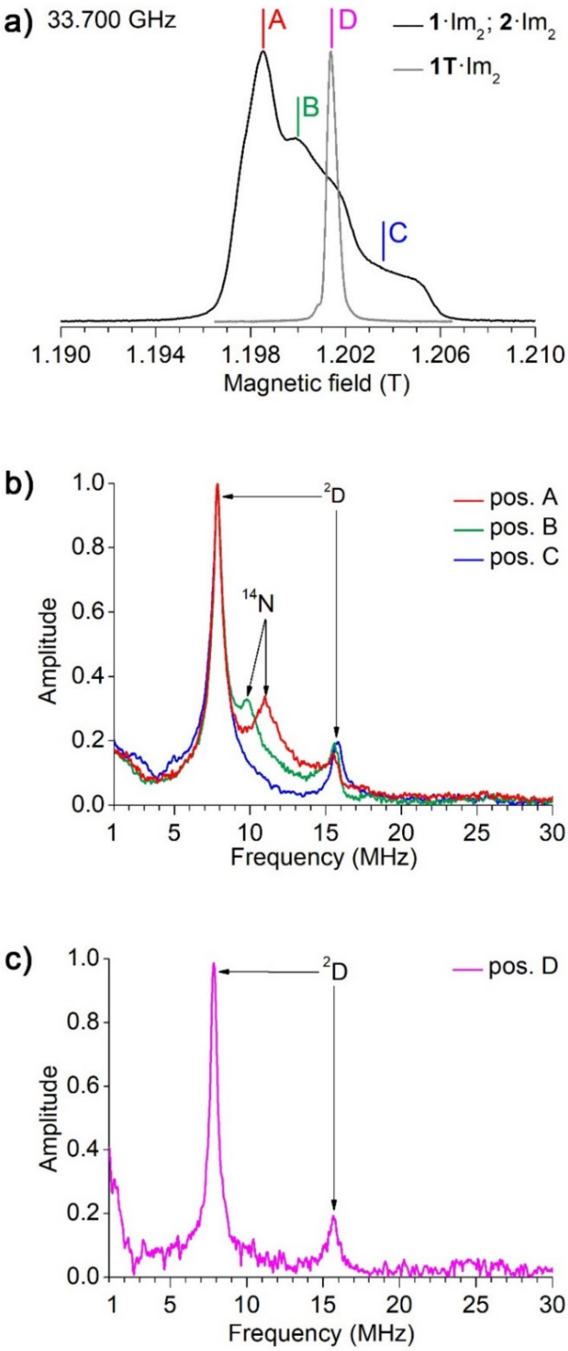

Figure S4. Q-band ESEEM experiments on the nitroxide centers of **1**·Im$_2$ and **1T**·Im$_2$ and the trityl center of **2**·Im$_2$. **a)** The Q-band echo-detected EPR spectra of nitroxide (black line) and trityl (gray line) spin centers. The detection positions in the ESEEM experiments are depicted by short bars and labeled with letters A-D. **b)** The ESEEM spectra of the nitroxide spin center in **1**·Im$_2$ and **2**·Im$_2$. **c)** The ESEEM spectra of the trityl spin center in **1T**·Im$_2$. The deuterium and nitrogen ESEEM peaks are marked by arrows.

6 Inversion recovery experiments on the ls Fe^{3+} center of **1**·Im₂, **2**·Im₂ and **1T**·Im₂

Inversion recovery measurements were carried out using the pulse sequence π-t-$\pi/2$-τ-π-τ-*echo*. The frequency of the microwave pulses was in resonance with either the g_1-, g_2- or g_3-component of the ls Fe^{3+} spectrum. The lengths of the $\pi/2$- and π-pulses were 12 and 24 ns, respectively. Depending on the detection position, τ was set either to 468 ns (for g_{eff} = 2.91), 320 ns (for g_{eff} = 2.28) or 304 ns (for g_{eff} = 2.66). The initial value of t was 500 ns, which was incremented 512 times with a step that depended on the temperature. It ranged from 200 ns at 16 K to 8 μs at 6 K. The shot repetition time was adjusted in accordance with the t increment and ranged from 120 μs to 4.1 ms.

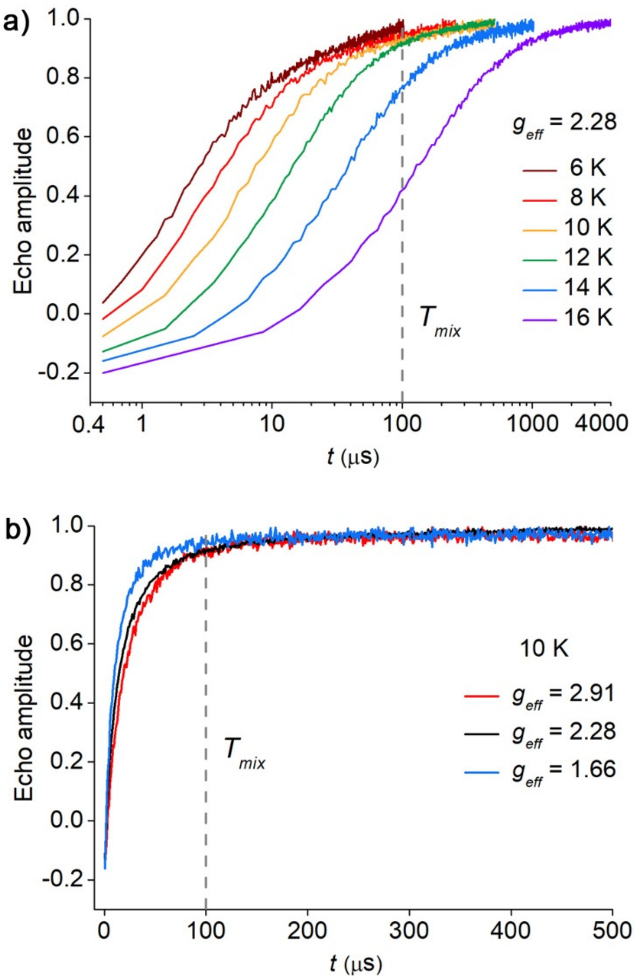

Figure S5. Inversion recovery of the ls Fe^{3+} magnetization in **1**·Im_2, **2**·Im_2 and **1T**·Im_2 at Q-band. **a)** The temperature dependence of the inversion recovery for the ls Fe^{3+} centers recorded at an effective g-factor of 2.28. **b)** The dependence of the inversion recovery on the effective g-factor of the ls Fe^{3+} at which the inversion recovery experiment was performed (T = 10 K). The value of T_{mix} is depicted as a dashed line.

7 RIDME experiments on 1·Im_2, 2·Im_2 and 1T·Im_2

7.1 Choosing the value of T_{mix}

The optimal way to perform a RIDME experiment on ls Fe^{3+}/nitroxide and ls Fe^{3+}/trityl spin pairs implies the detection of the echo signal from the slow-relaxing nitroxide or trityl spins, while the fast-relaxing Fe^{3+} spins are flipped stochastically in the interval T_{mix}. In this case, the length of T_{mix} should to be in agreement with the T_1 relaxation rate of the Fe^{3+} spins. Such an agreement can be readily obtained by performing an inversion recovery experiment on the Fe^{3+} spin centers and setting T_{mix} to a time, at which almost complete recovery of the Fe^{3+} longitudinal magnetization took place. The fact that the relaxation rate of the ls Fe^{3+} spin centers is temperature dependent makes it possible to adjust the optimal value of T_{mix} with temperature. This, in turn, can be employed to ensure that the length of T_{mix} fulfills a few additional criteria. One of such criteria is the need of T_{mix} to be significantly longer than any other inter-pulse time intervals in the RIDME pulse sequence, which helps to avoid unwanted flipping of the Fe^{3+} spins in these intervals. Moreover, T_{mix} has to be much shorter than the spin diffusion rates of the detected nitroxide or trityl spins, so that the spin diffusion does not significantly reduce the SNR of the RIDME signal. For the model compounds **1**·Im_2, **2**·Im_2 and **1T**·Im_2, the latter two criteria are well-fulfilled for $T_{mix} = 100$ μs, which fits to the T_1 relaxation rate of the Fe^{3+} spins for all three model systems at a temperature of 10 K (Figure S5a). It is also worth mentioning that the ls Fe^{3+} centers of all three model compounds showed a rather weak orientation dependence of their transversal relaxation time T_1 (Figure S5b) as compared to the strong orientation dependence found for the corresponding hs Fe^{3+} center.[3] This fact simplifies the task of choosing the optimal value of T_{mix} and ensures the same modulation depth for different orientations of the ls Fe^{3+} ions with respect to $\boldsymbol{B_0}$.

7.2 ESEEM suppression scheme

Since ESEEM penetrates into the RIDME time traces and causes unwanted artifacts in the corresponding spectra, additional steps have to be taken during the RIDME measurements to suppress them. As the deuterium ESEEM peak at 7.8 MHz is the most intense one for all three model compounds (see Figure S4), it was the main target of the ESEEM suppression in the present study. The suppression of this peak was done in accordance to the method proposed by Yulikov and co-workers.[4] The idea behind this method is to average the RIDME time trace over one period of ESEEM modulation. To do this, the initial values of intervals τ_1 and τ_2 in the RIDME sequence were incremented consecutively 16 times with a step of 8 ns,

resulting in 256 individual RIDME time traces. The sum of these time traces yielded the averaged time trace with the ESEEM artifacts suppressed.

7.3 Phase cycling

In order to avoid an overlap of the detected reversed virtual echo with unwanted echoes, the RIDME time traces were acquired using 16-step phase cycling (Scheme S2). As compared to the usual 8-step phase cycling,[5] in which the phases of 1st, 3rd and 4th m.w. pulses are varied, the 16-step phase cycling scheme introduces an additional phase cycle [(+*x*) (-*x*)] for the 2nd m.w. pulse. This additional phase cycle allowed more efficient suppression of the unwanted reversed virtual echo, which is created by the 1st, 2nd, 4th and 5th m.w. pulses and which interferes with the detected echo at time $t = \tau_1$.

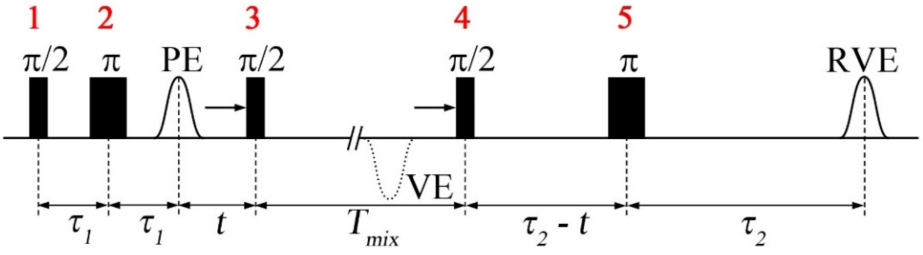

ϕ(pulse 1)	ϕ(pulse 2)	ϕ(pulse 3)	ϕ(pulse 4)	ϕ(pulse 5)	Detection
+*x*	+*x*	+*x*	+*x*	+*x*	+
+*x*	+*x*	-*x*	-*x*	+*x*	+
+*x*	+*x*	+*y*	+*y*	+*x*	+
+*x*	+*x*	-*y*	-*y*	+*x*	+
-*x*	+*x*	+*x*	+*x*	+*x*	-
-*x*	+*x*	-*x*	-*x*	+*x*	-
-*x*	+*x*	+*y*	+*y*	+*x*	-
-*x*	+*x*	-*y*	-*y*	+*x*	-
+*x*	-*x*	+*x*	+*x*	+*x*	+
+*x*	-*x*	-*x*	-*x*	+*x*	+
+*x*	-*x*	+*y*	+*y*	+*x*	+
+*x*	-*x*	-*y*	-*y*	+*x*	+
-*x*	-*x*	+*x*	+*x*	+*x*	-
-*x*	-*x*	-*x*	-*x*	+*x*	-
-*x*	-*x*	+*y*	+*y*	+*x*	-
-*x*	-*x*	-*y*	-*y*	+*x*	-

Scheme S2. 16-step phase cycle used for 5-pulse RIDME sequence.

8 Signal-to-noise ratio of RIDME time traces

The signal-to-noise ratio (SNR) of the background-corrected RIDME time traces was determined by means of the program SnrCalculator (free available at https://github.com/dinarabdullin/SnrCalculator). In this program, the SNR is determined as a ratio between the modulation depth of the RIDME time trace and the standard deviation of noise.[6] To extract the noise from the experimental RIDME time traces, first these time traces are interpolated using the Savitzky-Golay filter and then the result of interpolation is subtracted from the time traces.

Table S2. SNR of the RIDME time traces of **1**·Im_2, **2**·Im_2 and **1T**·Im_2.

**Parameter**	**1**·Im_2	**2**·Im_2	**1T**·Im_2
modulation depth (%)	29	40	42
SNR (unitless)	905	1616	3520
SNR ($1 / h^{1/2}$)	59	64	70
SNR (*dB*)	447	798	1739
SNR ($dB / h^{1/2}$)	29	31	35

9 DipFit analysis of the RIDME time traces of 1·Im$_2$, 2·Im$_2$ and 1T·Im$_2$

Due to the inversion symmetry of the magnetic tensors (spin Hamiltonian), the 180° rotation of one of the *g*-tensor principal axes of the ls Fe^{3+} center does not influence the shape of the RIDME time trace.[7] This leads to four symmetry-related sets of angular parameters ‹ξ› and ‹φ›, which provide identical RIDME time traces und thus can not be distinguished in RIDME data analysis. Tables S3, S4 and S5 list the symmetry-related sets of angular parameters for the model compounds **1**·Im$_2$, **2**·Im$_2$ and **1T**·Im$_2$, respectively.

Table S3. Symmetry-related sets of RIDME-derived parameters of **1**·Im$_2$.

**Transformation**	**Fitting parameter**					
	‹r› (nm)	Δr (nm)	‹ξ› (°)	Δξ (°)	‹φ› (°)	Δφ (°)
Fitting result	2.48 ± 0.03	0.05 ± 0.05	69 ± 21	24 ± 24	27 ± 27	20 ± 20
180° rotation about g_{xx}			111 ± 21		333 ± 6	
180° rotation about g_{yy}			111 ± 21		153 ± 6	
180° rotation about g_{zz}			69 ± 21		207 ± 6	

Table S4. Symmetry-related sets of RIDME-derived parameters of **2**·Im$_2$.

**Transformation**	**Fitting parameter**					
	‹r› (nm)	Δr (nm)	‹ξ› (°)	Δξ (°)	‹φ› (°)	Δφ (°)
Fitting result	2.44 ± 0.02	0.05 ± 0.04	84 ± 23	23 ± 23	30 ± 30	13 ± 17
180° rotation about g_{xx}			96 ± 23		330 ± 30	
180° rotation about g_{yy}			96 ± 23		150 ± 30	
180° rotation about g_{zz}			84 ± 23		210 ± 30	

Table S5. Symmetry-related sets of RIDME-derived parameters of **1T**·Im$_2$.

**Transformation**	**Fitting parameter**					
	‹r› (nm)	Δr (nm)	‹ξ› (°)	Δξ (°)	‹φ› (°)	Δφ (°)
Fitting result	2.64 ± 0.01	0.06 ± 0.02	71 ± 19	3 ± 20	57 ± 7	30 ± 9
180° rotation about g_{xx}			109 ± 19		303 ± 7	
180° rotation about g_{yy}			109 ± 19		123 ± 7	
180° rotation about g_{zz}			71 ± 19		237 ± 7	

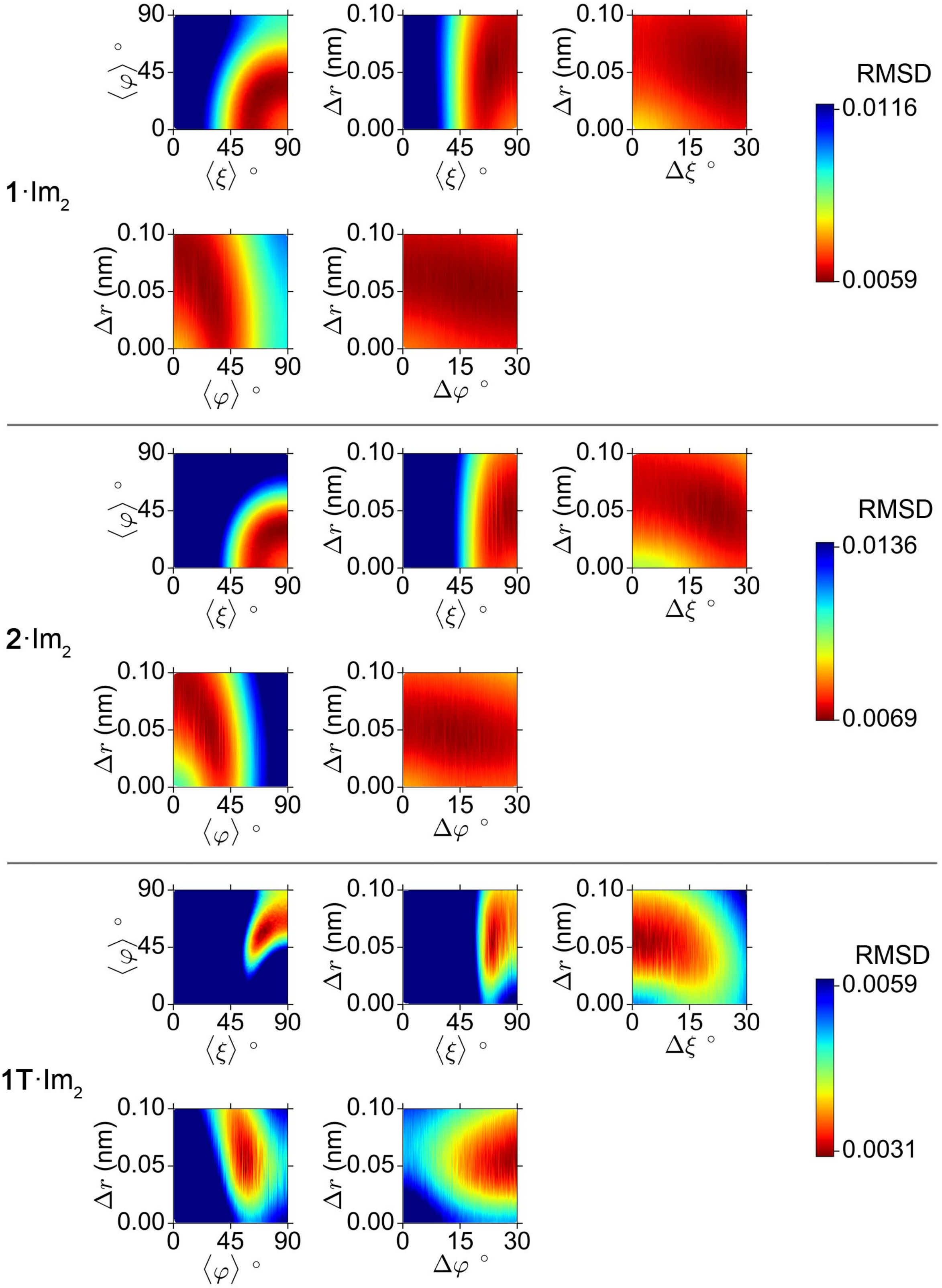

Figure S6. Dependencies of the RMSD between the experimental and simulated RIDME time traces on different pairs of fitting parameters.

10 DipFit analysis of the RIDME time trace of the P450cam mutant C58R1

The RIDME time trace of the cytochrome P450cam mutant C58R1 (Figure S7) was taken from our previous paper.[8] This time trace was measured at the Q-band frequency on the C58R1 sample with the protonated buffer. The DipFit procedure, which was applied to fit the RIDME time trace of C58R1, was identical to the one described in the Experimental section of the main text. An important difference to the case of the model compounds was the use of other g-values for the ls Fe^{3+} center: $g_{xx} = 1.91$, $g_{yy} = 2.25$, $g_{zz} = 2.42$.[8] The results of the DipFit analysis are summarized in Figure S7, Figure S8 and Table S6.

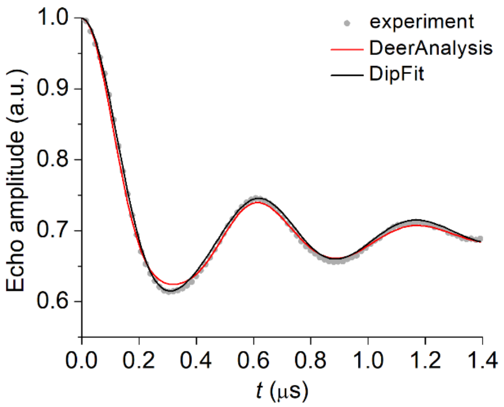

Figure S7. Background-corrected RIDME time trace (gray dots) of the P450cam mutant C58R1 is shown together with its fits obtained by means of DipFit (black line) and DeerAnalysis (red) programs.

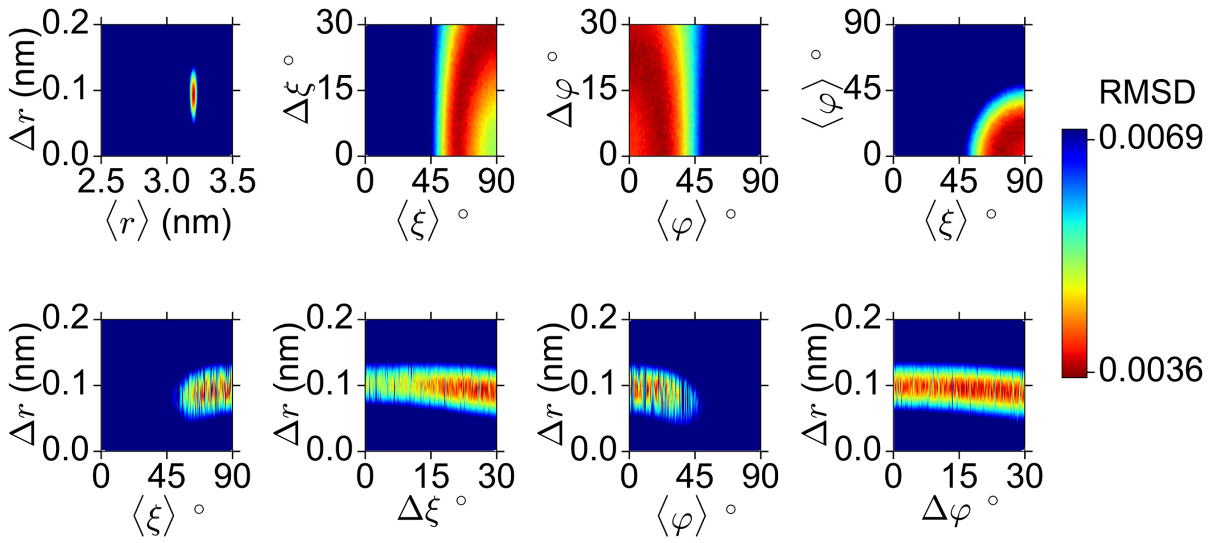

Figure S8. Dependencies of the RMSD between the experimental and simulated RIDME time traces on the mean values and standard deviations of the fitting parameters r, ξ and φ.

Table S6. RIDME-derived parameters of C58R1.

Transformation	Fitting parameter					
	⟨r⟩ (nm)	Δr (nm)	⟨ξ⟩ (°)	$\Delta\xi$ (°)	⟨φ⟩ (°)	$\Delta\varphi$ (°)
Fitting result	3.20 ± 0.01	0.09 ± 0.01	77 ± 18	26 ± 26	11 ± 18	20 ± 20
180° rotation about g_{xx}			103 ± 18		349 ± 18	
180° rotation about g_{yy}			103 ± 18		169 ± 18	
180° rotation about g_{zz}			77 ± 18		191 ± 18	

11 MD simulations for 1·Im$_2$, 2·Im$_2$ and 1T·Im$_2$

The structure optimization and MD simulations for model compounds **1**·Im$_2$, **2**·Im$_2$ and **1T**·Im$_2$ were done using the stand-alone program *xtb*.[9] Evaluation of the MD trajectories was performed with the program TRAVIS.[10] Owing to the bi-radical electronic structure and the large molecular size of the model compounds, the semi-empirical tight-binding method GFN2-xTB/GBSA[11–13] was applied. The modeling began with the generation of initial structures for all three compounds, followed by the optimization of these structures at the GFN2-xTB/GBSA(THF) level of theory. Next, a conformer/rotamer ensemble (CRE) was generated for each initial structure using the search algorithm *crest*.[14] The CRE generation was conducted using THF as a solvent, which was simulated by the implicit GBSA solvation model.[15] For each CRE, the energetically lowest conformer was determined and later used as a starting point for molecular dynamic (MD) simulations. The MD simulations were carried out using the implicit solvent GBSA (THF) and a temperature of 298 K. The SHAKE[16] algorithm was used to constrain all bonds. The time window was set to 1 ns, the time step to 4 fs, and the equilibration phase to 10 ps.

To enable the comparison of the MD results with the structural information from RIDME, the distributions $P(r)$, $P(\xi)$ and $P(\varphi)$ were calculated based on the MD trajectories. The values of r were determined as the distance between the Fe atom and the center of the N-O bond of the nitroxide radical or the central C atom of the trityl radical. To determine the angular parameters ξ and φ, the orientation of the ls Fe^{3+} g-tensor relative to tetraphenylporphyrin (TPP) had to be defined. Here, this orientation was set to the one reported for $Fe(TPP)(4\text{-}MeIm)_2^+$.[17] Thus, the g_{zz}-axis of ls Fe^{3+} was orthogonal to the TPP plane and aligned with the Fe-N(imidazole) bond. The corresponding g_{xx}- and g_{yy}-axes were aligned with two orthogonal Fe-N(porphyrin) bonds within the TPP plane. Based on this definition of the g-axes, ξ was determined as the angle between the g_{zz}-axis of ls Fe^{3+} and the inter-spin vector $\boldsymbol{r}$. The angle between the g_{xx}-axis and the projection of $\boldsymbol{r}$ on the TPP plane yielded the value of φ. The resulting MD distributions of r, ξ and φ are summarized in Figure S9. Increasing the MD time window from 500 ps to 1 ns did not lead to any significant change in the predicted distributions (Figure S9), suggesting that the system may be regarded as equilibrated. To allow direct comparison to the RIDME results, the calculated distributions were approximated by Gaussians (Figure S10) and the mean values and their standard deviation were calculated for each distribution (Table 1 in the main text).

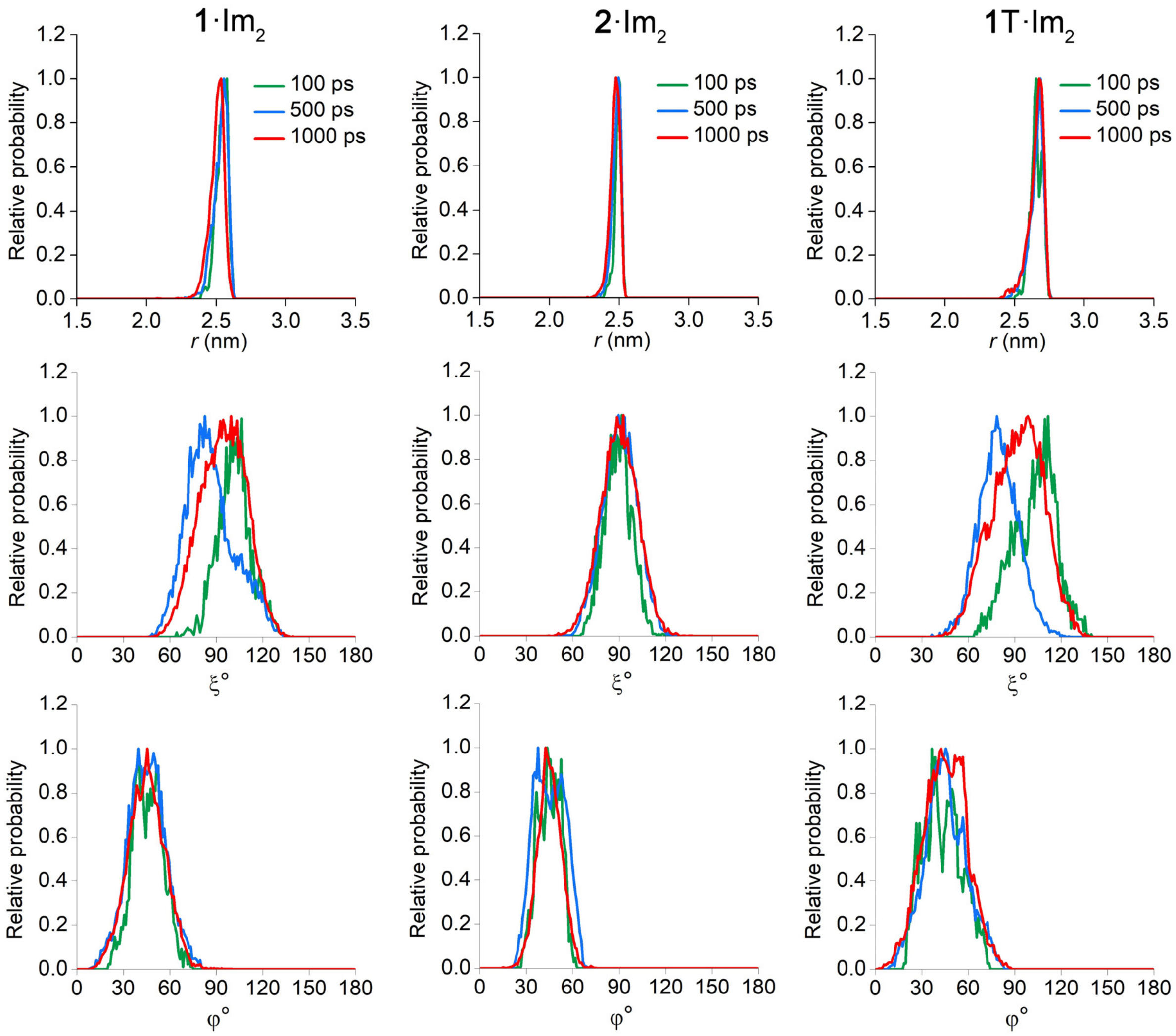

Figure S9. The MD predictions of the distributions $P(r)$, $P(\xi)$ and $P(\varphi)$ for the MD time window of 100 ps (green), 500 ns (blue) and 1 ns (red).

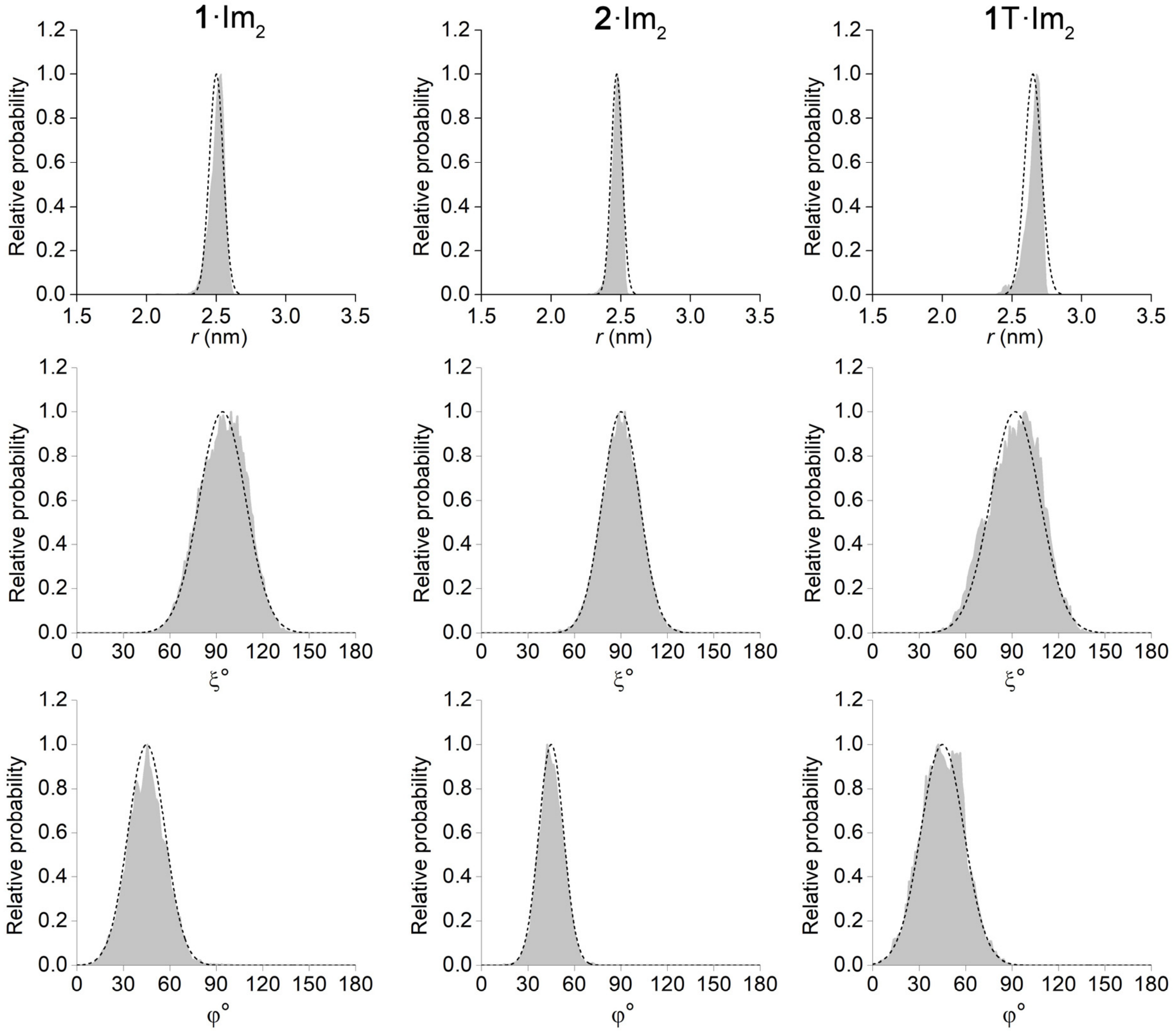

Figure S10. The MD predictions (gray shades) of the distributions $P(r)$, $P(\xi)$ and $P(\varphi)$ in the model compounds **1**·Im$_2$, **2**·Im$_2$ and **1T**·Im$_2$. The fits of all distributions by Gaussians is depicted by black dashed lines. The parameter of the Gaussians are listed in Table 1 of the main text.

12 Optimized structure of 1·Im$_2$

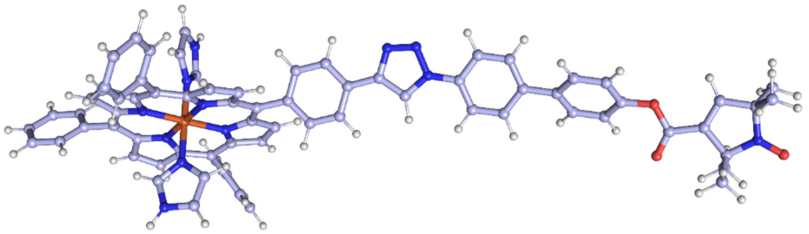

Figure S11. The ball-and-stick model of the optimized structure of **1**·Im$_2$. Atom colors: hydrogen – gray, carbon – light blue, nitrogen – blue, oxygen – red, iron – orange.

Coordinates:

C	9.0412882095	-0.8778968290	-0.1546458267
C	9.5851035568	0.4043541572	-0.1215458661
C	8.8160102991	1.5671492657	-0.1687173700
N	7.4584552954	1.6345565673	-0.0965809828
C	7.1353038864	2.9559563739	-0.1785624841
C	5.8583194149	3.4919363046	-0.0244253908
C	4.7189510263	2.7159933598	0.1868361936
N	4.6481786189	1.3586447814	0.1193023457
C	3.3554995585	1.0275909332	0.3930123602
C	2.8100600854	-0.2526456844	0.3158935406
C	3.5527460983	-1.3851647321	-0.0181322707
N	4.9042196801	-1.4460702394	-0.1587191226
C	5.1986726153	-2.7366666636	-0.4802283406
C	6.4783611164	-3.2769313772	-0.5855594003
C	7.6442758350	-2.5301211683	-0.4182263177
N	7.7188797745	-1.1777177820	-0.2883794346
FE	6.1813507496	0.0907602425	-0.1061408889
N	6.0555490604	0.2497481110	-2.1110751481
C	7.0506345306	0.3891225780	-2.9570363959
N	6.5952643534	0.4810078446	-4.2193391655
C	5.2353953507	0.3947141664	-4.1715213680
C	4.9143818760	0.2511343900	-2.8549997842
H	3.9524146616	0.1477067382	-2.3973846785
H	4.6239844678	0.4397706439	-5.0483561375
H	7.1528999275	0.5949593165	-5.0508237850
H	8.0859565888	0.4285978062	-2.6852103316
N	6.3088035698	-0.0578723290	1.8994952513
C	6.2890590492	0.9263077736	2.7691925393
N	6.3789843632	0.4552210292	4.0258365419
C	6.4608263753	-0.9035530910	3.9491534671
C	6.4162383779	-1.2079930122	2.6217179399

H	6.4559958993	-2.1642526361	2.1427061021
H	6.5421798566	-1.5261114306	4.8154344492
H	6.3827088073	1.0022145193	4.8720642432
H	6.2089281454	1.9647075442	2.5188254410
C	8.9541393097	-3.1084234256	-0.3170686677
C	9.8199268986	-2.0838330344	-0.1381640659
H	10.8818091948	-2.1318155365	0.0008981965
H	9.1688239091	-4.1582755642	-0.3533854100
C	6.6168443564	-4.7270048065	-0.8317650680
C	6.0994988025	-5.6567997579	0.0657408493
C	6.2405682276	-7.0121965250	-0.1703893567
C	6.8947211879	-7.4567434717	-1.3071926747
C	7.4130900367	-6.5386457315	-2.2053574894
C	7.2797712438	-5.1829639512	-1.9678324545
H	7.6804150813	-4.4648643583	-2.6690078327
H	7.9239473859	-6.8803520668	-3.0933429680
H	7.0019805907	-8.5148458643	-1.4916445975
H	5.8395753870	-7.7239399734	0.5358248543
H	5.5974296063	-5.3107296000	0.9578945874
C	3.9893218915	-3.4994418303	-0.6101344041
C	2.9685112164	-2.6643314927	-0.3079402623
H	1.9178542789	-2.8780237938	-0.3024276818
H	3.9371329726	-4.5302816851	-0.8996947247
C	1.3650555261	-0.4256259206	0.5588930039
C	0.4172079654	0.2269830035	-0.2256291849
C	-0.9322894022	0.0507086785	0.0057873642
C	-1.3678151930	-0.7805807239	1.0361553659
C	-0.4198461284	-1.4341601408	1.8238884402
C	0.9263894271	-1.2626735923	1.5837924107
H	1.6562288183	-1.7690805727	2.1987630618
H	-0.7664004164	-2.0745682910	2.6196531211
C	-2.7766851291	-0.9880060210	1.3083620787
C	-3.9065009401	-0.4936656147	0.6812327405
N	-4.9459069043	-1.0319031670	1.3669519753
N	-4.4702399908	-1.8054764061	2.3515884361
N	-3.2031203544	-1.7851231207	2.3249425261
C	-6.3221194888	-0.8830672682	1.1711211875
C	-6.8363470384	-0.0292816803	0.2011424867
C	-8.2035384282	0.0847530995	0.0438719069
C	-9.0824969517	-0.6476343279	0.8389170401
C	-8.5520282251	-1.4989336844	1.8081729000
C	-7.1892824828	-1.6158662373	1.9779327145
H	-6.7709022481	-2.2662865009	2.7290960890
H	-9.2164853029	-2.0582265423	2.4487805101

C	-10.5355326174	-0.5267684622	0.6590203936
C	-11.3545577336	-1.6535826024	0.7207087860
C	-12.7179712832	-1.5444920845	0.5423557261
C	-13.2859462015	-0.2983037019	0.3063369708
C	-12.4909071839	0.8369207579	0.2564850899
C	-11.1247579932	0.7133988792	0.4263027389
H	-10.5104533336	1.6005597263	0.4018808858
H	-12.9302524684	1.8061592157	0.0857112736
O	-14.6722363491	-0.2652942162	0.1861566843
C	-15.2797061980	0.5525331782	-0.6823690670
O	-14.7262271141	1.2948327854	-1.4525973776
C	-16.7489591048	0.4083753088	-0.5868166725
C	-17.4349928519	-0.3588852169	0.2600692532
C	-18.9100997891	-0.2354975186	0.0479248850
N	-18.9750676337	0.7217066880	-1.0512010976
O	-20.0419028528	1.1098774448	-1.5479073532
C	-17.6665712395	1.1745085217	-1.5033604311
C	-17.4790273816	0.8021950949	-2.9770222675
H	-16.5089374216	1.1544341398	-3.3145592737
H	-18.2621355216	1.2687078023	-3.5693907826
H	-17.5301549720	-0.2765295928	-3.1092453610
C	-17.5605615124	2.6910547403	-1.3164033388
H	-16.5897857336	3.0274214492	-1.6676727893
H	-18.3445705136	3.1804902295	-1.8887141602
H	-17.6701437301	2.9564118094	-0.2669298546
C	-19.6411609356	0.3436137597	1.2645571710
H	-19.6480984665	-0.3794371327	2.0747270620
H	-20.6657673013	0.5791182286	0.9869255415
H	-19.1518199631	1.2524131993	1.6074230001
C	-19.5591931565	-1.5527508528	-0.3923789841
H	-19.5699618770	-2.2578256568	0.4334570233
H	-20.5809335847	-1.3587562809	-0.7099039290
H	-19.0097629890	-1.9904036372	-1.2227591887
H	-17.0202963369	-1.0003546802	1.0157887607
H	-13.3551932060	-2.4140763182	0.5800842009
H	-10.9161131763	-2.6260648933	0.8847726388
H	-8.5946537993	0.7359069516	-0.7231187689
H	-6.1816429404	0.5477601781	-0.4327629029
H	-4.0278373783	0.1566732486	-0.1569461666
H	-1.6506931530	0.5603509943	-0.6186720652
H	0.7461386332	0.8643735091	-1.0340512381
C	2.6006985584	2.2082797455	0.7028490158
C	3.4430835989	3.2579056484	0.5589246497
H	3.2362796801	4.2989957665	0.7115688212

H	1.5700186618	2.2233944117	0.9973652210
C	5.7006178092	4.9607351069	-0.0350765175
C	4.8848656559	5.5679343911	-0.9860274526
C	4.7320204696	6.9420774401	-1.0045333961
C	5.3840633512	7.7277957262	-0.0686413310
C	6.1916325504	7.1321024766	0.8856831083
C	6.3514538440	5.7584250152	0.9018637820
H	6.9755468572	5.2941770261	1.6523270399
H	6.6983092675	7.7397399267	1.6207270611
H	5.2622783652	8.8002149312	-0.0820072158
H	4.1017094627	7.4019117536	-1.7512095749
H	4.3803514354	4.9530786080	-1.7175230558
C	8.3212579603	3.7413174674	-0.3683618941
C	9.3658638596	2.8805921372	-0.3470283050
H	10.4085508231	3.1019533494	-0.4636255929
H	8.3433265371	4.8042424684	-0.5063875328
C	11.0548419084	0.5499315636	-0.0869165964
C	11.8492826416	0.0363483653	-1.1081645975
C	13.2238794878	0.1835543748	-1.0692682855
C	13.8238655737	0.8408976654	-0.0082956266
C	13.0415342919	1.3555930188	1.0121727935
C	11.6664920297	1.2156990761	0.9717896904
H	11.0545794919	1.6127560781	1.7689903586
H	13.5046998399	1.8682960327	1.8420898188
H	14.8969650137	0.9527616796	0.0224528860
H	13.8289135127	-0.2154095531	-1.8698808688
H	11.3817516254	-0.4700092709	-1.9408053851

13 Optimized structure of 2·Im$_2$

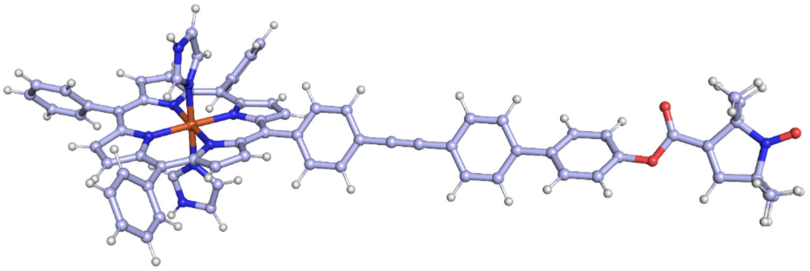

Figure S12. The ball-and-stick model of the optimized structure of **2**·Im$_2$. Atom colors: hydrogen – gray, carbon – light blue, nitrogen – blue, oxygen – red, iron – orange.

Coordinates:

FE	-5.8420560134	0.0182963455	-0.0086127061
N	-7.2095393603	1.4809277763	0.0795054969
C	-6.9708929417	2.8196648127	0.0244272326
C	-5.7485112222	3.4247128430	-0.2649750324
C	-4.5529681224	2.7208069541	-0.3919611763
N	-4.3887857264	1.3841364559	-0.1881513208
C	-3.0534793205	1.1430129179	-0.2869834271
C	-2.4272569549	-0.0926537023	-0.1248091988
C	-3.1210207918	-1.2933846136	0.0176818952
N	-4.4729007999	-1.4449048011	-0.0487316078
C	-4.7119055886	-2.7834819641	0.0144272555
C	-5.9615154321	-3.3992933239	-0.0446817054
C	-7.1661176550	-2.6989800228	-0.0252732861
N	-7.3018652396	-1.3512837034	0.1235161531
C	-8.6384787717	-1.1092021979	0.2148648761
C	-9.2368020680	0.1370406813	0.3989542387
C	-8.5343655593	1.3397601557	0.3640898243
C	-9.1372173159	2.6286319796	0.5544103854
C	-8.1690013216	3.5476369204	0.3327643459
H	-8.2436220658	4.6155866418	0.3918007320
H	-10.1591183212	2.7977131962	0.8308861688
C	-10.6997883818	0.1797742878	0.6003709956
C	-11.5236749429	0.8544952639	-0.2962719782
C	-12.8930138781	0.8846070492	-0.1049768712
C	-13.4574806096	0.2462489481	0.9866465522
C	-12.6456952286	-0.4267382294	1.8846244197
C	-11.2769973311	-0.4639907691	1.6914452181
H	-10.6415295334	-0.9842834444	2.3936443404
H	-13.0809131513	-0.9246204432	2.7383461276
H	-14.5260426391	0.2725926380	1.1365505679

H	-13.5218008602	1.4075434320	-0.8102223314
H	-11.0850186115	1.3475667703	-1.1523300376
C	-9.3722515837	-2.3339173052	0.0699052782
C	-8.4583893767	-3.3195760466	-0.0900980963
H	-8.6344982522	-4.3653658024	-0.2472064321
H	-10.4413545983	-2.4161612962	0.0701427815
C	-6.0041239670	-4.8752231358	-0.0932356014
C	-5.4077910934	-5.5585049138	-1.1495783626
C	-5.4450867001	-6.9397807125	-1.2016053514
C	-6.0705950812	-7.6578475084	-0.1958708087
C	-6.6611941393	-6.9871101733	0.8619959117
C	-6.6310406966	-5.6053660791	0.9126402210
H	-7.0864183901	-5.0833917689	1.7424354390
H	-7.1466731054	-7.5423669498	1.6508245926
H	-6.0970460419	-8.7361663987	-0.2358067243
H	-4.9843511490	-7.4581560919	-2.0293735107
H	-4.9245887164	-4.9960700846	-1.9355556808
C	-3.4775225554	-3.4972217104	0.1780070315
C	-2.4906950130	-2.5710709227	0.1914022981
H	-1.4378438555	-2.7300561190	0.3168003548
H	-3.3894801056	-4.5601607912	0.2886003492
C	-0.9526100963	-0.1278826092	-0.1472967968
C	-0.2180698247	0.5947573375	0.7920087984
C	1.1602398344	0.5712902277	0.7792315994
C	1.8421282113	-0.1723711789	-0.1875978355
C	1.1094509570	-0.8927332226	-1.1342809411
C	-0.2690382491	-0.8710863872	-1.1085046621
H	-0.8304697875	-1.4219934002	-1.8496305963
H	1.6331124825	-1.4639830352	-1.8866245854
C	3.2510553973	-0.1914822416	-0.2084188834
C	4.4592581066	-0.2038895753	-0.2249476001
C	5.8682962351	-0.2148187155	-0.2408118002
C	6.5609143605	-0.9339367030	-1.2179626149
C	7.9392852392	-0.9428335701	-1.2260382168
C	8.6650636243	-0.2322215576	-0.2697068021
C	7.9680882401	0.4870508224	0.7016406563
C	6.5900674548	0.4953350925	0.7219077891
H	6.0575788394	1.0483180427	1.4818070233
H	8.5167558667	1.0244095601	1.4610232734
C	10.1335244374	-0.2399488295	-0.2828862273
C	10.8532092982	0.9239280189	-0.0219524316
C	12.2350551972	0.9286287740	-0.0285940349
C	12.9158929657	-0.2509526323	-0.2888730938
C	12.2180442164	-1.4216533054	-0.5576751751

C	10.8384895702	-1.4114058434	-0.5572937453
H	10.3008694974	-2.3280393551	-0.7471094828
H	12.7677400288	-2.3276932806	-0.7598581767
O	14.3062707189	-0.3262993122	-0.3541219720
C	15.0761967921	0.3279662596	0.5220508189
O	14.6816786667	0.9913725649	1.4467958218
C	16.5082679744	0.1119877362	0.2160249275
C	17.0260200449	-0.5673831459	-0.8069095730
C	18.5211985566	-0.5600572809	-0.7757464804
N	18.7916563155	0.2264932627	0.4228911363
O	19.9363548924	0.4764066837	0.8229899339
C	17.5858069443	0.6868587558	1.0974707253
C	17.5736258779	2.2182184163	1.1279700179
H	18.4557553308	2.5757676012	1.6531403083
H	17.5749663679	2.6218428312	0.1175671973
H	16.6805070567	2.5574904295	1.6439399899
C	17.5506729271	0.1229012135	2.5209442538
H	17.5375925490	-0.9648634977	2.5020139589
H	16.6565404319	0.4786735726	3.0240264592
H	18.4313021251	0.4569550475	3.0637877036
C	19.1200884330	-1.9607082278	-0.6039809037
H	20.1846843623	-1.8719411506	-0.4009875714
H	18.6456407080	-2.4797393596	0.2257563100
H	18.9768807578	-2.5412263818	-1.5104766355
C	19.1393542235	0.1439272141	-1.9892163902
H	18.6791669372	1.1175842175	-2.1414411081
H	18.9950091183	-0.4567883390	-2.8822816455
H	20.2045862285	0.2808640984	-1.8190086473
H	16.4749843999	-1.0709614467	-1.5797337498
H	12.7756358390	1.8403182961	0.1678544092
H	10.3258698732	1.8466892819	0.1666990177
H	8.4649895574	-1.4875044476	-1.9965764239
H	6.0060989160	-1.4800831865	-1.9668082833
H	1.7236322757	1.1273434168	1.5140439883
H	-0.7426637168	1.1675449982	1.5432817194
C	-2.3566017156	2.3547000029	-0.6152492790
C	-3.2889047346	3.3323681415	-0.6902273562
H	-3.1451629006	4.3666204676	-0.9330803846
H	-1.3011054336	2.4335945912	-0.7867819054
C	-5.7190165587	4.8955355978	-0.4005138690
C	-4.9527269074	5.6749756756	0.4615146713
C	-4.9356649225	7.0518912477	0.3332233701
C	-5.6782522365	7.6679269402	-0.6602270021
C	-6.4429563823	6.9002402153	-1.5228728531

C	-6.4673815501	5.5238673796	-1.3919988512
H	-7.0600026210	4.9228402453	-2.0667610613
H	-7.0229477764	7.3759022895	-2.2997749693
H	-5.6623111924	8.7425042292	-0.7609339054
H	-4.3417288252	7.6463427476	1.0115046019
H	-4.3797903951	5.1954576533	1.2423884933
N	-6.0127000091	-0.0446579011	-2.0184277761
C	-7.1235115419	-0.0036368105	-2.7183964952
N	-6.8619837732	-0.0875539763	-4.0350530974
C	-5.5098085050	-0.1869594736	-4.1786988494
C	-4.9953076651	-0.1588534681	-2.9172238044
H	-3.9748218177	-0.2101501333	-2.5988126095
H	-5.0359539029	-0.2663700683	-5.1346493641
H	-7.5383781590	-0.0809625358	-4.7818967981
H	-8.1074023399	0.0811711251	-2.3035119892
N	-5.6820216997	0.0680292967	2.0027418885
C	-5.7007076122	-0.9581986072	2.8224997847
N	-5.5749192797	-0.5525967673	4.0987586556
C	-5.4710671918	0.8068730496	4.0882640315
C	-5.5391034146	1.1784407519	2.7790160956
H	-5.4980831718	2.1572065309	2.3479587361
H	-5.3616817265	1.3836382866	4.9827163098
H	-5.5588620866	-1.1417500836	4.9160467527
H	-5.7986690576	-1.9816800354	2.5220351742

14 Optimized structure of 1T·Im$_2$

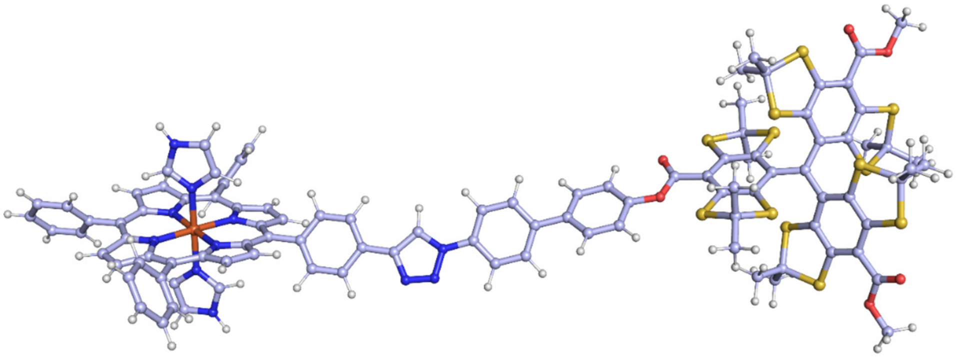

Figure S13. The ball-and-stick model of the optimized structure of **1T**·Im$_2$. Atom colors: hydrogen – gray, carbon – light blue, nitrogen – blue, oxygen – red, sulfur – yellow, iron – orange.

Coordinates:

C	14.3038601483	2.3811080705	1.4740992920
C	15.6033025601	2.7822573594	1.7752286665
C	16.7369179409	2.0584543188	1.4123727564
N	16.7493877655	0.8275802088	0.8318015107
C	18.0591070946	0.4671527543	0.7278975246
C	18.5363959451	-0.7564629708	0.2594286080
C	17.7164418801	-1.7506299888	-0.2742789991
N	16.3810752430	-1.6407060108	-0.5194948503
C	16.0017791608	-2.8110067963	-1.1017966581
C	14.7322682370	-3.1084398974	-1.5916776269
C	13.6338291295	-2.2635400039	-1.4497145144
N	13.5979856420	-1.1088231255	-0.7302930472
C	12.3181477100	-0.6488981776	-0.8079574790
C	11.8113522042	0.4750895926	-0.1551004925
C	12.5966203233	1.3478295301	0.5985878821
N	13.9535845401	1.3105047665	0.7097230946
FE	15.1705590874	-0.1524731694	0.0721126127
N	14.9054617513	-1.1205560458	1.8295562862
C	15.8379238531	-1.6405839284	2.5946712154
N	15.2970944384	-2.2181018073	3.6825562646
C	13.9456564222	-2.0548931678	3.6035426546
C	13.7172496629	-1.3712715526	2.4471874046
H	12.7902048868	-1.0445644972	2.0240049902
H	13.2758817343	-2.4247531628	4.3514153562
H	15.7953323548	-2.6880215379	4.4215579644
H	16.8881931181	-1.6159621640	2.3857464174
N	15.4347073594	0.8122150212	-1.6895622710
C	14.5161464268	1.4229204124	-2.4030124532

N	15.0489163819	1.9466668698	-3.5214818807
C	16.3802764464	1.6524338133	-3.5176214310
C	16.6053247683	0.9474485634	-2.3735253171
H	17.5196103939	0.5359257477	-1.9995532029
H	17.0406372472	1.9579344674	-4.3020014672
H	14.5574564564	2.4613525640	-4.2347985080
H	13.4805566202	1.4953567408	-2.1391712476
C	12.0686744637	2.4417188497	1.3636609741
C	13.1288798257	3.0821184054	1.9084118623
H	13.1242659852	3.9376852831	2.5544567575
H	11.0272522433	2.6700087926	1.4778045504
C	10.3682554564	0.7614870504	-0.2723156292
C	9.4190803622	-0.1643950133	0.1544156732
C	8.0699421045	0.1028529262	0.0389465947
C	7.6345761907	1.3052743382	-0.5153329564
C	8.5829696970	2.2347787687	-0.9421648952
C	9.9292438005	1.9667279970	-0.8181720597
H	10.6569242249	2.6909738780	-1.1560748066
H	8.2370298896	3.1624845390	-1.3702029779
C	6.2257197995	1.6137715310	-0.6622756759
C	5.0972165964	0.8823123952	-0.3367352208
N	4.0569571314	1.6694946125	-0.7071118398
N	4.5294706027	2.8098891957	-1.2255463224
N	5.7972911668	2.7865594051	-1.2017571576
C	2.6817200020	1.4330047657	-0.6179585503
C	2.1714062960	0.3470255102	0.0850703293
C	0.8063319903	0.1472367889	0.1402368646
C	-0.0752754115	1.0228259757	-0.4905123941
C	0.4516495986	2.1138230520	-1.1819504424
C	1.8129508669	2.3183603084	-1.2507570699
H	2.2285173920	3.1552999084	-1.7886725707
H	-0.2136752556	2.7941557393	-1.6910589225
C	-1.5258350292	0.8011986410	-0.4308230629
C	-2.0563359226	-0.4806074927	-0.5438631332
C	-3.4203899778	-0.7049450044	-0.5012509153
C	-4.2784478125	0.3729145026	-0.3356911813
C	-3.7656265502	1.6626786359	-0.2145635951
C	-2.4050059818	1.8718753828	-0.2652184386
H	-2.0158045124	2.8724367673	-0.1535330232
H	-4.4553517570	2.4805999262	-0.0791963861
O	-5.6585266376	0.3000630581	-0.2732730959
C	-6.3859904746	-0.8136717774	-0.3857170573
O	-5.9597248941	-1.9326781378	-0.5961057971
C	-7.8163750055	-0.5543118244	-0.2445851953

C	-8.3618729570	0.6717068235	0.1507204754
C	-9.7402558083	0.8287364014	0.2221421634
C	-10.6128971543	-0.2539058518	-0.0041937953
C	-10.0485523865	-1.4921032107	-0.3616301303
C	-8.6736277477	-1.6257637736	-0.5237945770
S	-8.0483458901	-3.1543527446	-1.0522074634
C	-9.6946391032	-3.8086677612	-1.6244082315
C	-9.7718951592	-5.2948649515	-1.3367243219
H	-9.6436657240	-5.4859914718	-0.2737211958
H	-8.9836468384	-5.8126028871	-1.8787062334
H	-10.7347678986	-5.6931125185	-1.6509818499
C	-9.8634112317	-3.4865685910	-3.0984053434
H	-9.0573858363	-3.9433093707	-3.6690804656
H	-10.8154747012	-3.8694769235	-3.4621215573
H	-9.8385070536	-2.4095358349	-3.2588001694
S	-11.0006707450	-2.9258680643	-0.6706066190
C	-12.0348203265	-0.0917300915	0.1301374966
C	-12.6673544957	1.0831187035	-0.3858082637
C	-13.7188082371	1.7116771091	0.3163736825
C	-14.3345100801	2.8514969840	-0.1946900466
C	-13.8905870797	3.4208501594	-1.3927522791
C	-12.8397241981	2.8206429873	-2.0959570268
C	-12.2548743031	1.6592410707	-1.6091353435
S	-11.1085559521	0.8723823479	-2.6765862653
C	-10.7330358933	2.3774308647	-3.6692201103
C	-9.5775540228	3.1151771725	-3.0166512291
H	-9.8294778574	3.3807508841	-1.9911321149
H	-9.3613316148	4.0266970960	-3.5702324586
H	-8.6886748633	2.4862291110	-3.0054394321
C	-10.4457781149	1.9862871913	-5.1050233101
H	-10.2587285415	2.8780229880	-5.6992620401
H	-9.5688445496	1.3434680686	-5.1495802199
H	-11.2927657809	1.4541393931	-5.5315592481
S	-12.2448456812	3.4509068138	-3.6057002558
C	-14.5749611899	4.6350059433	-1.8245619698
O	-15.4259135597	5.1751993462	-1.1448423174
O	-14.1855728340	5.0806412939	-3.0179199581
C	-14.8151269476	6.2652993475	-3.4855415609
H	-15.8931132553	6.1125371005	-3.5662389738
H	-14.3815758476	6.4709350418	-4.4605155527
H	-14.6263184955	7.0907694952	-2.7963918220
S	-15.6228254479	3.5752916649	0.7070903562
C	-15.7835833674	2.1755135254	1.9280948651
C	-16.0311139075	2.7404451485	3.3124491867

H	-15.2088050083	3.3854685674	3.6127415510
H	-16.9479170756	3.3257233082	3.3087620033
H	-16.1296771823	1.9358634101	4.0385765414
C	-16.8955230228	1.2513871700	1.4626293740
H	-17.8346825415	1.7991580591	1.4233264264
H	-16.6745138681	0.8670247621	0.4682485730
H	-17.0024806539	0.4109660374	2.1466195407
S	-14.2038444509	1.2275756373	1.9173677698
C	-12.8012986078	-1.1313282091	0.7489742480
C	-12.3048388784	-1.8228969445	1.8729763580
C	-13.0189704570	-2.8743628599	2.4398104817
C	-14.2611146809	-3.2474415773	1.9156063084
C	-14.7644750736	-2.5802813628	0.7934662332
C	-14.0578478527	-1.5218647265	0.2363093188
S	-14.8027188245	-0.7367352616	-1.1414976436
C	-15.9186430834	-2.1438877968	-1.5564824582
C	-17.2104270958	-1.6132070418	-2.1446053760
H	-17.0064728816	-1.0627054832	-3.0608282404
H	-17.7070900110	-0.9495979274	-1.4404122721
H	-17.8787063198	-2.4406468265	-2.3727369828
C	-15.1893695776	-3.0914619991	-2.4914891640
H	-15.8139237227	-3.9569730162	-2.7036995387
H	-14.9545619146	-2.5851847358	-3.4262002958
H	-14.2598473199	-3.4335249732	-2.0380285362
S	-16.2711468132	-3.0224998014	0.0369715766
C	-14.9746354775	-4.2995796161	2.6325292501
O	-14.5128618325	-4.8361673306	3.6210704601
O	-16.1704655601	-4.5891513562	2.1230022330
C	-16.9136743146	-5.5991243251	2.7909317532
H	-17.0672103112	-5.3252122090	3.8365646401
H	-17.8632120544	-5.6688780813	2.2671708654
H	-16.3795990245	-6.5504664613	2.7500984866
S	-12.3574885896	-3.6844703805	3.8195847655
C	-10.6856631566	-2.8641533052	3.7101523528
C	-9.7462467496	-3.7855747192	2.9512571892
H	-10.1416238262	-3.9909665436	1.9578704815
H	-9.6452721340	-4.7274353000	3.4862983699
H	-8.7633240114	-3.3285180564	2.8473324025
C	-10.1909249660	-2.5395343770	5.1053691828
H	-10.8797650240	-1.8625038652	5.6050213110
H	-10.1194976053	-3.4550530026	5.6882869662
H	-9.2086497202	-2.0728493255	5.0613650304
S	-10.8805878319	-1.3112911220	2.7414956880
S	-10.3162478178	2.4266087074	0.6603868107

C	-8.7412155132	2.9501934475	1.4612430596
C	-8.5553634646	4.4448777636	1.2948614095
H	-8.5327412791	4.7127729375	0.2407871623
H	-7.6185324549	4.7540853998	1.7535627826
H	-9.3738073814	4.9782428512	1.7740147748
C	-8.7581146437	2.5148967861	2.9148231514
H	-7.8076475125	2.7573140416	3.3866404525
H	-9.5586152523	3.0259642552	3.4465726339
H	-8.9245635464	1.4409352462	2.9917194193
S	-7.3855498078	2.0520861103	0.5763182389
H	-3.8071534800	-1.7053760698	-0.6005346468
H	-1.3951789120	-1.3209396571	-0.6927236419
H	0.4171431892	-0.6887186359	0.7014945767
H	2.8283283798	-0.3382153417	0.5976562821
H	4.9762264931	-0.0879590650	0.0925888654
H	7.3513073279	-0.6265027247	0.3815095616
H	9.7483279433	-1.0963608288	0.5915184487
C	11.5359797060	-1.5084120776	-1.6505014871
C	12.3522106857	-2.5127363959	-2.0458714106
H	12.1222233938	-3.3366725210	-2.6922784960
H	10.5081039426	-1.3494688808	-1.9113087469
C	14.5404141260	-4.3872937252	-2.3058770164
C	13.6419866361	-5.3360095468	-1.8255756554
C	13.4653946661	-6.5349736279	-2.4917707950
C	14.1777614361	-6.7995410242	-3.6496568987
C	15.0706251610	-5.8596702426	-4.1370942775
C	15.2540226068	-4.6628035758	-3.4689225967
H	15.9450429018	-3.9256004827	-3.8518355336
H	15.6261452080	-6.0600332110	-5.0413117323
H	14.0375379306	-7.7347519245	-4.1703189730
H	12.7698462325	-7.2658307963	-2.1063044635
H	13.0918319727	-5.1305658060	-0.9184305793
C	17.1186993329	-3.7097913674	-1.1751440651
C	18.1838684549	-3.0511023434	-0.6627032354
H	19.1877961201	-3.4080157388	-0.5417259658
H	17.0808867993	-4.7115847851	-1.5552287574
C	19.9893614703	-1.0156533629	0.3329087937
C	20.7208581038	-1.2668688077	-0.8246565069
C	22.0810592749	-1.5064366715	-0.7568556028
C	22.7282322095	-1.5049535567	0.4675653547
C	22.0084424326	-1.2590780211	1.6248462128
C	20.6492378160	-1.0130996805	1.5586873682
H	20.0876972676	-0.8249711189	2.4630560594
H	22.5077899233	-1.2588214864	2.5824478473

H	23.7896071422	-1.6942860797	0.5198055814
H	22.6382313423	-1.6945148124	-1.6625768913
H	20.2171715622	-1.2625240110	-1.7807885063
C	18.9018104229	1.5272211766	1.2042068455
C	18.0805634648	2.5142628097	1.6309101850
H	18.3495460937	3.4683615616	2.0393112404
H	19.9739343164	1.5156594494	1.1942446022
C	15.7908628361	4.0471872746	2.5151596207
C	16.4136900561	4.0515188691	3.7600829945
C	16.5865133958	5.2351972449	4.4539748119
C	16.1463780225	6.4309049430	3.9111691059
C	15.5290591329	6.4367523568	2.6714447362
C	15.3489541563	5.2533514817	1.9788923813
H	14.8739509135	5.2575596954	1.0083391860
H	15.1868841676	7.3669674074	2.2425264704
H	16.2842481036	7.3547796170	4.4522176450
H	17.0663191498	5.2250744039	5.4214927202
H	16.7509228266	3.1170142955	4.1852226042

References

[1] S. Stoll, A. Schweiger, *J. Magn. Reson.* **2006**, *178*, 42–55.

[2] M. Florent, I. Kaminker, V. Nagarajan, D. Goldfarb, *J. Magn. Reson.* **2011**, *210*, 192–199.

[3] D. Abdullin, H. Matsuoka, M. Yulikov, N. Fleck, C. Klein, S. Spicher, G. Hagelueken, S. Grimme, A. Luetzen, O. Schiemann, *Chem. - A Eur. J.* **2019**, DOI 10.1002/chem.201900977.

[4] K. Keller, A. Doll, M. Qi, A. Godt, G. Jeschke, M. Yulikov, *J. Magn. Reson.* **2016**, *272*, 108–113.

[5] S. Milikisyants, F. Scarpelli, M. G. Finiguerra, M. Ubbink, M. Huber, *J. Magn. Reson.* **2009**, *201*, 48–56.

[6] T. Bahrenberg, Y. Yang, D. Goldfarb, A. Feintuch, *Magnetochemistry* **2019**, *5*, 20.

[7] D. Abdullin, G. Hagelueken, R. I. Hunter, G. M. Smith, O. Schiemann, *Mol. Phys.* **2015**, *113*, 544–560.

[8] D. Abdullin, F. Duthie, A. Meyer, E. S. Müller, G. Hagelueken, O. Schiemann, *J. Phys. Chem. B* **2015**, *119*, 13539–13542.

[9] Please contact xtb@thch.uni–bonn.de to obtain the program.

[10] M. Brehm, B. Kirchner, *J. Chem. Inf. Model.* **2011**, *51*, 2007–2023.

[11] S. Grimme, C. Bannwarth, P. Shushkov, *J. Chem. Theory Comput.* **2017**, *13*, 1989–2009.

[12] C. Bannwarth, S. Ehlert, S. Grimme, *J. Chem. Theory Comput.* **2019**, *15*, 1652–1671.

[13] S. Grimme, C. Bannwarth, S. Dohm, A. Hansen, J. Pisarek, P. Pracht, J. Seibert, F. Neese, *Angew. Chemie Int. Ed.* **2017**, *56*, 14763–14769.

[14] S. Grimme, *J. Chem. Theory Comput.* **2019**, *15*, 2847–2862.

[15] W. Clark Still, A. Tempczyk, R. C. Hawley, T. Hendrickson, *J. Am. Chem. Soc.* **1990**, *112*, 6127–6129.

[16] J. P. Ryckaert, G. Ciccotti, H. J. C. Berendsen, *J. Comput. Phys.* **1977**, *23*, 327–341.

[17] E. Vinck, S. Van Doorslaer, *Phys. Chem. Chem. Phys.* **2004**, *6*, 5224–5330.
